# Repurposing of Ibrutinib and Quizartinib as potent inhibitors of necroptosis

**DOI:** 10.1038/s42003-023-05353-5

**Published:** 2023-09-23

**Authors:** Fangmin Huang, Jiankun Liang, Yingying Lin, Yushi Chen, Fen Hu, Jianting Feng, Qiang Zeng, Zeteng Han, Qiaofa Lin, Yan Li, Jingyi Li, Lanqin Wu, Lisheng Li

**Affiliations:** 1 The School of Basic Medical Sciences, Fujian Medical University, Fuzhou, China; 2 Key Laboratory of Ministry of Education for Gastrointestinal Cancer, Fujian Medical University, 1 Xueyuan Road, Minhou, Fuzhou, China

**Keywords:** Small molecules, Kinases

## Abstract

Necroptosis is a form of regulated cell death that has been implicated in multiple diseases. TNF-induced necroptosis is regulated by necrosomes, complexes consisting of RIPK1, RIPK3 and MLKL. In this study, by screening of a small-compound library, we identified dozens of compounds that inhibited TNF-induced necroptosis. According to the mechanisms by which they inhibited necroptosis, these compounds were classified into different groups. We then identified Ibrutinib as an inhibitor of RIPK3 and found that Quizartinib protected against the TNF-induced systemic inflammatory response syndrome in mice by inhibiting the activation of RIPK1. Altogether, our work revealed dozens of necroptosis inhibitors, suggesting new potential approaches for treating necroptosis-related diseases.

## Abstract (web summary)

Screening a library of compounds identifies several inhibitors of TNF-induced necroptosis, including Ibrutinib as an inhibitor of RIPK3 and Quizartinib as an indirect RIPK1 kinase activity inhibitor.

## Introduction

Necroptosis is a form of regulated cell death that can be induced by different stimuli, including the activation of death receptors by cytokines or infectious pathogens^1^. Among the different inducers of necroptosis, tumor necrosis factor α (TNF)-mediated necroptosis has been intensively studied^1,2^. In the TNF-mediated necroptosis signaling pathway, receptor-interacting serine/threonine kinase 1 (RIPK1) recruits receptor-interacting serine/threonine kinase 3 (RIPK3) and forms RIPK1/RIPK3 amyloidal oligomers, leading to the activation and autophosphorylation of RIPK3^3–8^. RIPK3 further recruits and phosphorylates its downstream substrate mixed lineage kinase domain-like protein (MLKL)^9–12^. The phosphorylated MLKL oligomerize and target the plasma membrane to execute cell death^13–17^. Necroptosis has been implicated in various diseases, including atherosclerosis, bowel inflammation, acute pancreatitis and neurodegenerative diseases^18–21^. Therefore, interfering with necroptosis is an attractive strategy to cure necroptosis-related diseases.

Since the kinase activities of RIPK1 and RIPK3 are critical to TNF-induced necroptosis, inhibitors of RIPK1 and RIPK3 have been uncovered. Necrostatin-1 (NEC-1) and its derivatives specifically inhibit the kinase activity of RIPK1 and block TNF-induced necroptosis^22,23^. Several inhibitors, including GSK’840, GSK’843, and GSK’872, specifically targeting RIPK3 have also been identified^24,25^. Moreover, some FDA-approved drugs, including sorafenib, pazopanib, and regorafenib, have been shown to inhibit RIPK1 and/or RIPK3 in an off-target manner^26–29^. However, to our knowledge, no inhibitors of necroptosis are available for clinical usage to cure necroptosis-related diseases.

To identify additional necroptosis inhibitors, we screened a library of 1965 small-compounds for their ability to block TNF-induced necroptosis in L929 cells. Dozens of compounds, including some FDA-approved/clinical trial drugs, inhibited TNF-induced necroptosis. Interestingly, most of these compounds were kinase inhibitors. The potential mechanisms by which these compounds interfered with TNF-induced necroptosis were investigated by artificially inducing the dimerization/oligomerization of the RIPK1- or RIPK3-induced necroptosis system and detecting the phosphorylation levels of RIPK1 and RIPK3 induced by TNF. According to our results, we found that Ibrutinib is an inhibitor of RIPK3, inhibiting RIPK3 autophosphorylation and MLKL activation. We also found that Quizartinib inhibited the activation of RIPK1 and protected mice from TNF-induced systemic inflammatory response syndrome (SIRS).

## Results

### Identification of necroptosis inhibitors using a library of 1965 small-compounds

**Fig. 1:**
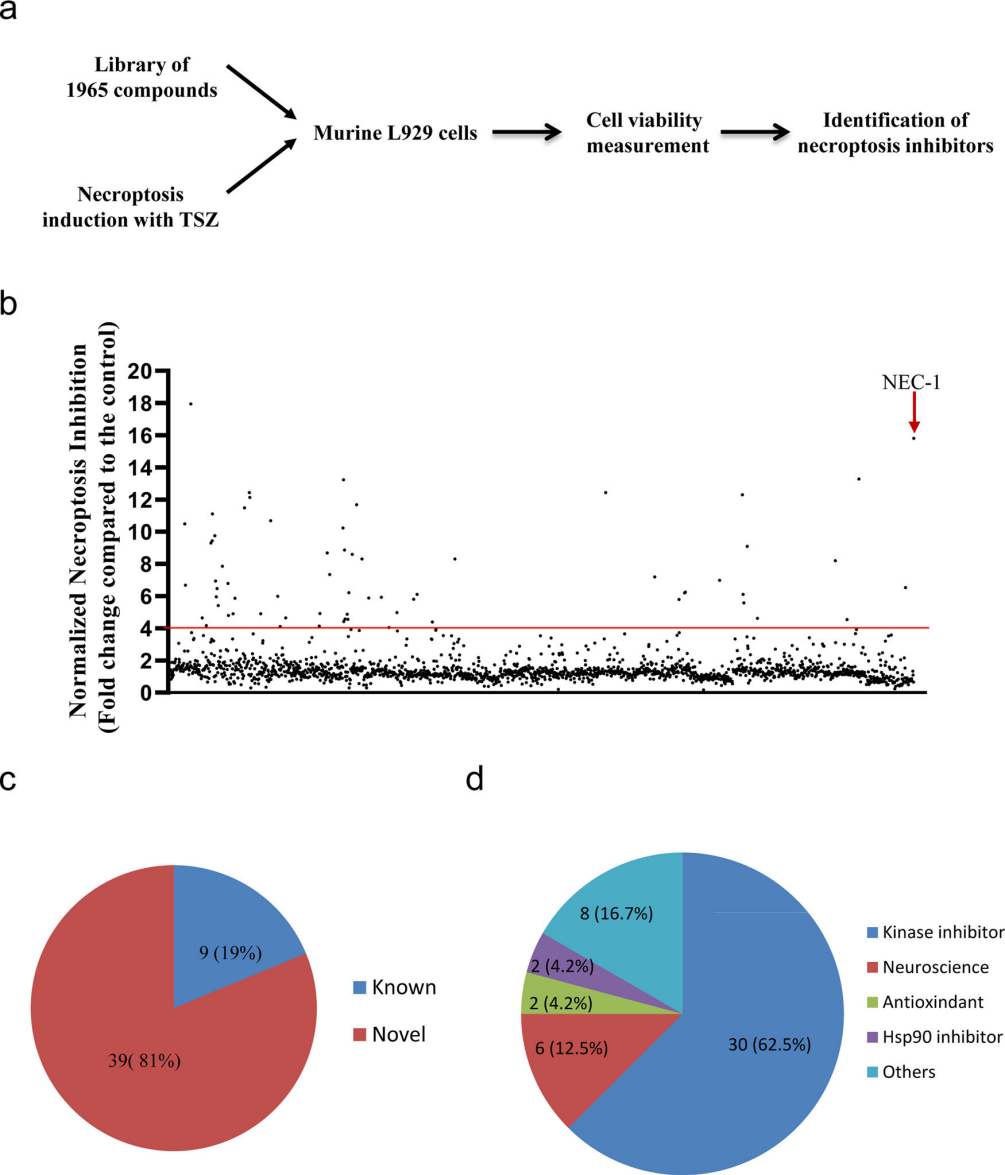
Identification of necroptosis inhibitors using a library of 1965 small-compounds. **a** Schematic overview of compound screen workflow. **b** L929 cells were pretreated with individual compound for 1 h following treatment with TNF (10 ng/ml), Smac mimetic (SM-164) (0.1 μM) and pan-caspase inhibitor z-VAD (10 μM) (TSZ) to induce necroptosis. Cell viabilities were determined with Cell Counting Kit-8 method and normalized to control (DMSO + TSZ). The concentration of individual compound is 15 μM. NEC-1 (20 μM) was chose as positive control. The red line indicated fold change compared to the control is 4. The screen data for each compound was singlicate. **c** Classified necroptosis inhibitors into known or novel inhibitors according to whether compounds had been reported to involved in necroptosis. **d** Classified necroptosis inhibitors into different groups according to their properties.

To discover novel inhibitors of necroptosis for potential clinical applications, a panel of 1965 compounds (APExBIO, Supplementary Data 1), including some FDA-approved/clinical trial drugs, were screened for their ability to block TNF-induced necroptosis in murine L929 cells (Fig. 1a). Cells were pretreated with individual compounds for 1 h before treatment with TNF, Smac mimetic (SM-164) and pan-caspase inhibitor z-VAD. The RIPK1 inhibitor NEC-1 was chosen as the positive control. As shown in Fig. 1b, we found dozens of compounds that inhibited TNF-induced necroptosis (fold change compared to the control >=4). We performed a secondary screening and confirmed that 48 compounds inhibited TNF-induced necroptosis (Supplementary Data 2). Among these, several compounds (9 of 48), such as HSP90 inhibitors AT13387 and Bardoxolone methyl, kinase inhibitors pazopanib, regorafenib, sorafenib and their analogs, had been previously reported^27–31^, while many compounds (39 of 48) were discovered to be necroptosis inhibitors (Fig. 1c and Supplementary Data 2). Furthermore, we found that more than one-half of the compounds were kinase inhibitors, which was consistent with TNF-induced necroptosis mediated by the kinase cascade RIPK1/RIPK3/MLKL (Fig. 1d and Supplementary Data 2). Interestingly, several compounds used in the neuroscience field were firstly found to be necroptosis inhibitors (Supplementary Data 2). Taken together, we discovered dozens of compounds that inhibited TNF-induced necroptosis.

### Classification of necroptosis inhibitors according to the mechanism studies

**Fig. 2:**
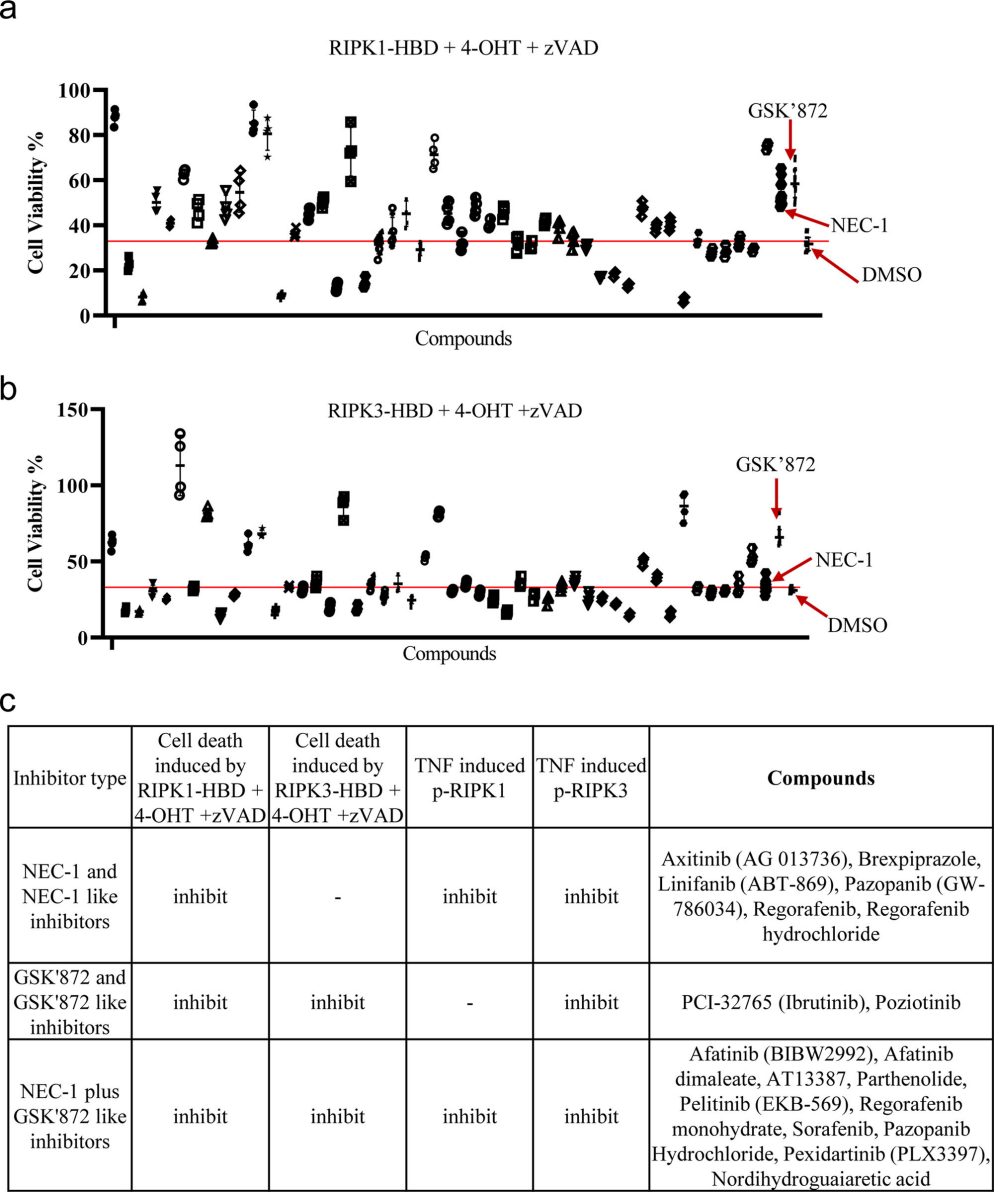
Classification of necroptosis inhibitors according to the mechanism studies. **a**, **b** Stable L929 cell line expressing RIPK1-HBD (**a**) or RIPK3-HBD (**b**) were pretreated with individual necroptosis inhibitor for 1 h following treatment with 4-OHT and z-VAD. The concentration of individual compound is 15 μM. NEC-1 (20 μM) and GSK’872 (10 μM) was chose as positive control. The red line indicated the value of cell viability of DMSO control. For compounds tested, *n* = 4 biologically independent samples. For NEC-1, GSK’872 and DMSO, *n* = 16 biologically independent samples. **c** Summary of classification of necroptosis inhibitors. Data shown are representative of three independent experiments. Means ± SD.

Then, we tried to dissect the potential mechanisms of how these compounds inhibit TNF-induced necroptosis. Artificially induced dimerization/oligomerization of proteins, such as RIPK1 and RIPK3, in the necroptosis signaling pathway induced necroptosis^32–35^. As shown in Fig. 2a, artificially induced dimerization/oligomerization of RIPK1 induced necroptosis, and this effect was reversed by the RIPK1 inhibitor NEC-1 and the RIPK3 inhibitor GSK’872. In contrast, necroptosis induced by the dimerization/oligomerization of RIPK3 was inhibited only by GSK’872, not by NEC-1 (Fig. 2b). When testing these compounds in necroptosis induced by the dimerization/oligomerization of RIPK1 or RIPK3, we found that 24 compounds and 19 compounds reduced necroptosis induced by the dimerization/oligomerization of RIPK1 and RIPK3, respectively (Fig. 2a, b and Supplementary Data 3). To further determine the potential mechanism by which these compounds inhibited TNF-induced necroptosis, we tested whether these compounds influenced the phosphorylation of RIPK1 and RIPK3. As shown in Supplementary Fig. 1a–e and Supplementary Data 3, we found that 30 compounds and 34 compounds reduced TNF-induced phosphorylation of RIPK1 and RIPK3, respectively. Then, we classified these compounds according their potential mechanisms. We noticed that NEC-1 inhibited TNF-induced phosphorylation of RIPK1 and RIPK3 and reduced necroptosis that had been induced by dimerization/oligomerization of RIPK1 but not RIPK3. Since some compounds, such as linifanib, functioned similarly to NEC-1, we termed these compounds NEC-1-like inhibitors (Fig. 2c and Supplementary Data 3). Interestingly, linifanib was recently reported to be RIPK1 kinase inhibitor^36,37^. Similarly, we classified the remaining compounds as GSK’872-like inhibitors, NEC-1 plus GSK’872-like inhibitors, or other types of inhibitors (Fig. 2c and Supplementary Data 3). We noticed that HSP90 inhibitor AT13387 were able to inhibit not only the phosphorylation of RIPK1 and RIPK3, but also necroptosis induced by dimerization/oligomerization of RIPK1 or RIPK3, which indicated AT13387 inhibited necroptosis at least at the level of RIPK1 and RIPK3. In contrast, most of neuroscience relative compounds that inhibit TNF-induced necroptosis did not interfere necroptosis induced by dimerization/oligomerization of RIPK1 or RIPK3, which implied these inhibitor might not play roles at the level of RIPK1 and RIPK3. In summary, the necroptosis inhibitors we identified inhibited TNF-induced necroptosis through different mechanisms.

### Ibrutinib inhibited TNF-induced phosphorylation of RIPK3 in different cell lines

**Fig. 3:**
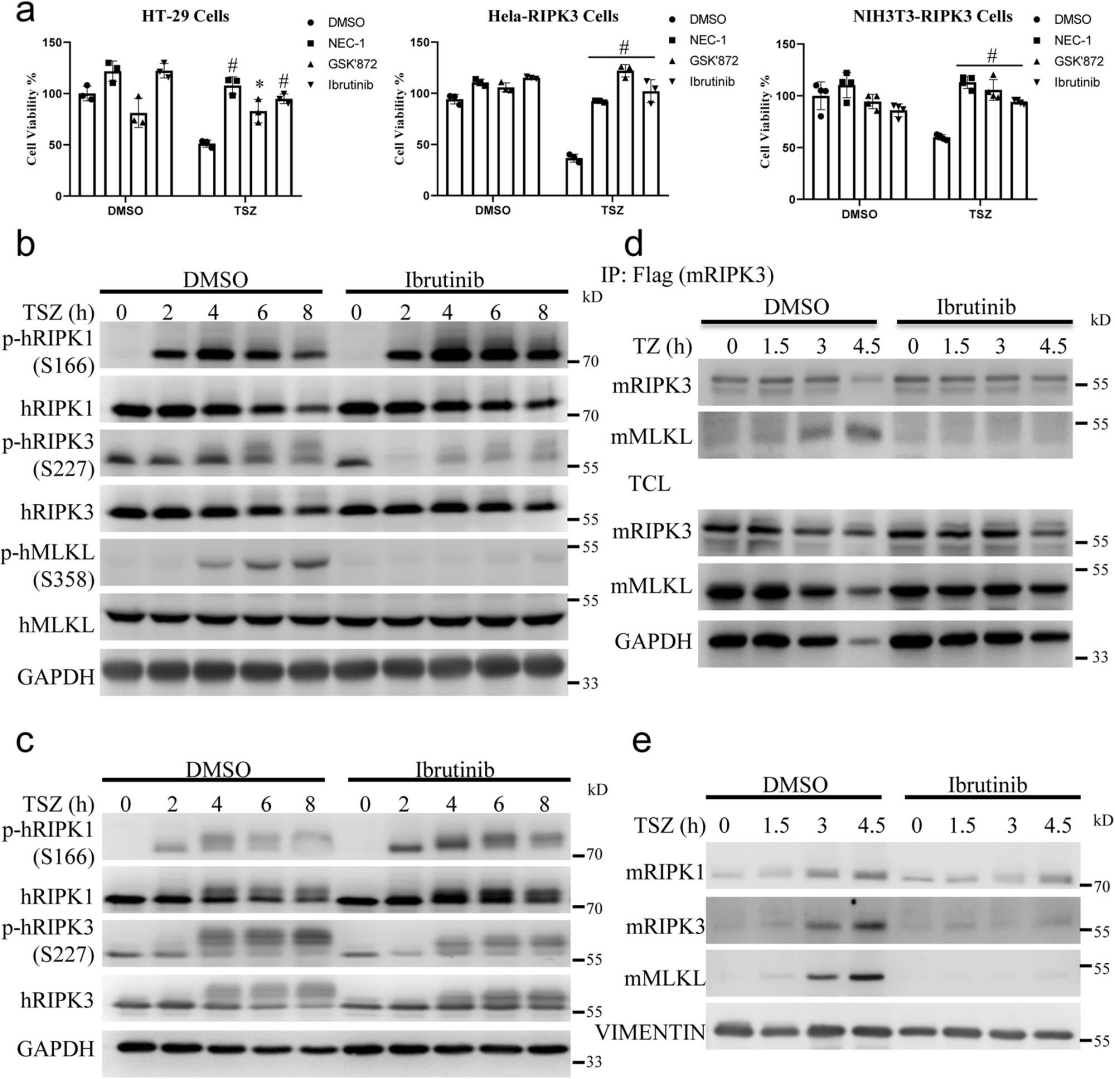
Ibrutinib inhibited TNF-induced phosphorylation of RIPK3 in different cell lines. **a** HT-29, Hela-RIPK3, and NIH3T3-RIPK3 cells were pretreated with NEC-1 (20 μM), GSK’872 (10 μM), Ibrutinib (15 μM) or vehicle for 1 h following treatment with TSZ. Cell viabilities were determined using CCK8 method. For HT-29 and Hela-RIPK3 cells, *n* = 3 biologically independent samples. For NIH3T3-RIPK3 cells, *n* = 4 biologically independent samples. **b**, **c** HT-29 cells (**b**) or Hela-RIPK3 cells (**c**) were pretreated Ibrutinib (15 μM) or vehicle for 1 h following treatment with TSZ for indicated time. Then cells were harvested and analyzed with the indicated antibodies. **d** Mouse RIPK3-flag reconstituted RIPK3-KO L929 cells were pretreated Ibrutinib (15 μM) or vehicle for 1 h following treatment with TSZ for indicated time. Then cells were harvested and immunoprecipitated with M2 (anti-flag) antibody. The total cell lysates (TCL) and the immunoprecipitates were immunoblotted with the indicated antibodies. **e** L929 cells were pretreated Ibrutinib (15 μM) or vehicle for 1 h following treatment with TSZ for indicated time. Then the cells were lysed with Triton X-100 lysis buffer. The insoluble fractions were collected and analyzed with the indicated antibodies. Data shown are representative of three independent experiments. Means ± SD. *p < 0.05, ^#^*p* < 0.01.

To validate the conclusions of the above analysis, we chose Ibrutinib for further investigation. According to the aforementioned data, we found that Ibrutinib is a GSK’872-like inhibitor that not only inhibited TNF or RIPK1 and RIPK3 dimerization/oligomerization-induced necroptosis but also specifically inhibited TNF-induced phosphorylation of RIPK3 in L929 cells. Ibrutinib inhibited necroptosis in a dose-dependent manner (Supplementary Fig. 2). We found that Ibrutinib sufficiently inhibited TNF-induced necroptosis in the HT-29 and HeLa-RIPK3 human cells and NIH3T3-RIPK3 murine cells (Fig. 3a). Consistent with this finding, Ibrutinib inhibited TNF-induced autophosphorylation of RIPK3 but not that of RIPK1 in HT-29 and HeLa-RIPK3 cells (Fig. 3b, c). Although the underlying mechanisms of mouse and human MLKL activation differ between species^12,38–40^, the kinase activity of RIPK3 is required for the recruitment of MLKL in both species^9,41^. Then we evaluated whether Ibrutinib influenced the interaction of RIPK3 and MLKL. As shown in Fig. 3d and Supplementary Fig. 3, Ibrutinib reduced the interaction of RIPK3 and MLKL induced by TNF in both human and mouse cell lines. Translocation of necrosomes into Triton X-100 or Nonidet P-40 insoluble pellets is important for necroptosis signal transduction^9,42^. As shown in Fig. 3e, we found that Ibrutinib reduced the translocation of RIPK1, RIPK3 and MLKL in L929 cells. Taken together, Ibrutinib inhibited the autophosphorylation of RIPK3 kinase, the interaction of RIPK3 and MLKL, and the translocation of necrosomes and thus inhibited TNF-induced necroptosis.

### RIPK3 is a target of Ibrutinib

**Fig. 4:**
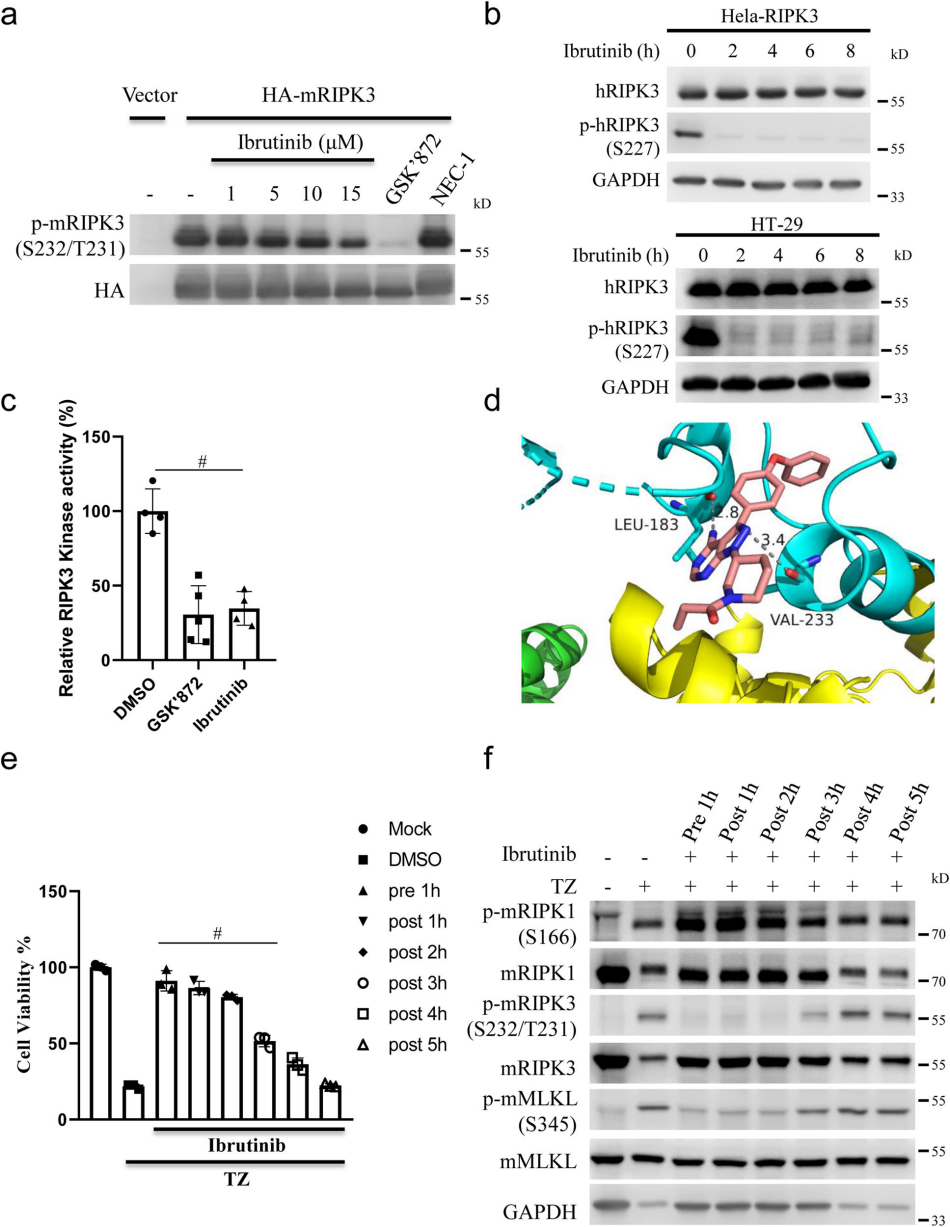
RIPK3 is a target of Ibrutinib. **a** Plasmid expressing HA-mRIPK3 or Vector was transfected into 293T cells. Then the cells were treated with indicated concentrations of Ibrutinib, GSK’872 (10 μM), NEC-1 (20 μM) for 20 h. The cells were harvested and analyzed with indicated antibodies. **b** Hela-RIPK3 or HT-29 was treated with Ibrutinib (15 μM) for the indicated times. Then the cells were harvested and analyzed with indicated antibodies. **c** In vitro ADP-Glo kinase assay using recombinant hRIPK3 protein. Recombinant hRIPK3 was incubated with DMSO, GSK’872 (10 μM) or Ibrutinib (10 μM). For DMSO and Ibrutinib, *n* = 4 biologically independent samples. For GSK’872, *n* = 5 biologically independent samples. **d** Molecular Docking simulation result of Ibrutinib and RIPK3 using RIPK3 structure (PDB code: 7MX3)^38^. **e** L929 cells were treated with Ibrutinib (15 μM) before or after challenging with TZ. Then cell viabilities were determined using CCK8 method. *n* = 3 biologically independent samples. **f** L929 cells were treated with Ibrutinib (15 μM) before or after challenging with TZ. Then cells were harvested and analyzed with indicated antibodies. Data shown are representative of three independent experiments. Means ± SD. ^#^*p* < 0.01.

Ibrutinib is a small-molecule covalent inhibitor of Bruton tyrosine kinase (BTK) and has been approved by the Food and Drug Administration (FDA) for the treatment of lymphoma, including chronic lymphocytic leukemia^43,44^. To explore the role of BTK in regulating necroptosis, we tested whether different BTK inhibitors inhibited necroptosis. As shown in Supplementary Fig. 4a, neither BIIB068 nor RN486 inhibited TNF-induced necroptosis. Moreover, unlike *RIPK3*, we failed to detected the expression of *BTK* mRNA in L929 cells (Supplementary Fig. 4b), which indicated that BTK was not involved in TNF-induced necroptosis in L929 cells. As mentioned above, we classified Ibrutinib as a GSK’872-like inhibitor, and then, we wondered whether Ibrutinib also targets RIPK3, similar to GSK’872. As shown in Fig. 4a, we found that Ibrutinib, although less efficiently than GSK’872, reduced the autophosphorylation level of RIPK3 when RIPK3 was overexpressed. Specifically, Ibrutinib did not influence the phosphorylation of RIPK1 when RIPK1 was overexpressed (Supplementary Fig. 5). Moreover, Ibrutinib inhibited the phosphorylation of RIPK3 at the basal level in HT-29 and HeLa-RIPK3 cells (Fig. 4b). To verify whether Ibrutinib directly inhibits RIPK3 kinase activity, we performed an in vitro kinase assay and found that Ibrutinib inhibited RIPK3 kinase activity (Fig. 4c). We noticed that a crystal structure of human RIPK3 complexed with specific RIPK3 inhibitor GSK’843 had been reported (PDB code: 7MX3)^38^. According to the crystal structure, GSK’843 bound to RIPK3 through multiple amino acid residues including Lys50 which is important for RIPK3 kinase activity^45^ (Supplementary Fig. 6a). To further understand the mechanism of Ibrutinib in inhibition of RIPK3 kinase, both Ibrutinib and GSK’872 were docked with the RIPK3 (PDB code: 7MX3)^38^. We found that Ibrutinib binds to the residues Leu183 and Val233 of RIPK3 with hydrogen bonds, while GSK’872, similar to GSK’843, binds to multiple amino acid residues including Lys50 (Fig. 4d and Supplementary Fig. 6b). Mutation of either Leu183 or Val233 of RIPK3 to alanine reduced autophosphorylation level of RIPK3 (Supplementary Fig. 7), which indicated that Leu183 and Val233 are important for the kinase activity of RIPK3. We proposed that mutation of Leu183 or Val233 might influence the ability of Ibrutinib in inhibition of RIPK3kinase activity. As shown in Supplementary Fig. 7, Ibrutinib is less sufficient in inhibition of autophosphorylation level of Leu183Ala (reduced to 47%) or Val233Ala (reduced to 54%) RIPK3 when compared to wildtype RIPK3 (reduced to 32%). Moreover, according to docking result, we noticed that the acrylamide warhead of Ibrutinib was not involved in the interaction between Ibrutinib and RIPK3. Consistently, an Ibrutinib analogs (Ibrutinib N1), which is lack of acrylamide warhead, inhibited TNF-induced necroptosis similar to Ibrutinib (Supplementary Fig. 8a, b). The impairment of RIPK3 kinase activity by GSK’872 was reported to induce apoptosis^25^. In contrast to GSK’872, Ibrutinib did not induce apoptosis, but inhibited TNF-induced necroptosis at the same concentration (Supplementary Fig. 9a, b). We proposed that the difference in inducing apoptosis between Ibrutinib and GSK’872 might due to their different binding modes in RIPK3. Since phosphorylation of RIPK3 is an intermediate or late event in the TNF-induced necroptosis signaling pathway, we proposed that Ibrutinib might prevent cell death even when cells are pretreated with TNF. As shown in Fig. 4e, Ibrutinib significantly protected cells from necroptosis even after 3 h of pre-treatment with TNF. Moreover, this outcome corresponded with the phosphorylation levels of RIPK3 and MLKL (Fig. 4f). Taken together, our data suggested that RIPK3 is a target of Ibrutinib.

### Quizartinib inhibited necroptosis through regulating RIPK1 kinase activity

**Fig. 5:**
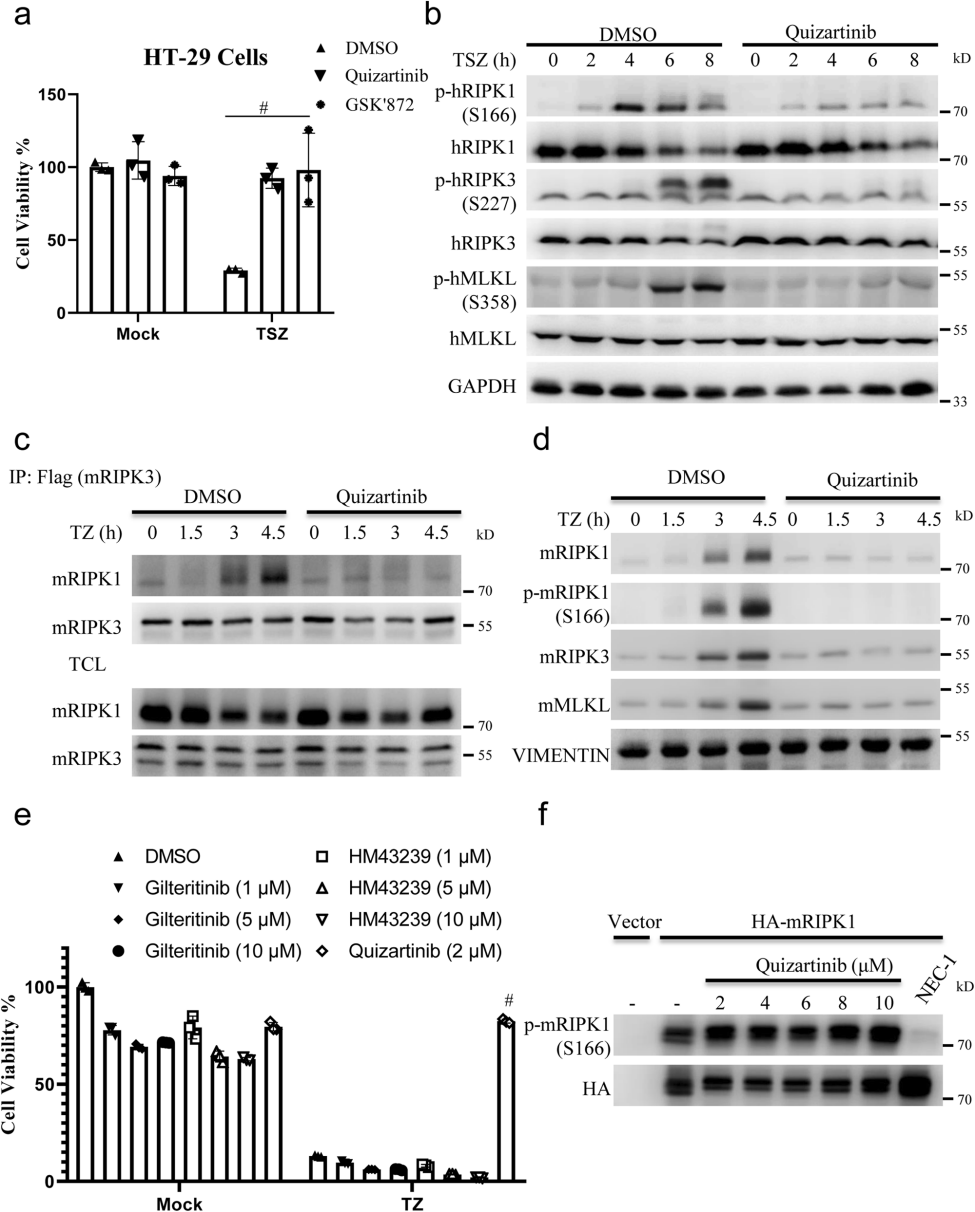
Quizartinib inhibited necroptosis through indirectly regulating RIPK1 kinase activity. **a** HT-29 cells were pretreated with vehicle, GSK’872 (10 μM), Quizartinib (10 μM) for 1 h following treatment with TSZ. Then the cell viabilities were determined using CCK8 method. *n* = 3 biologically independent samples. **b** HT-29 cells were pretreated with vehicle or Quizartinib (10 μM) for 1 h following treatment with TSZ for the indicated times. Then the cells were harvested and analyzed with the indicated antibodies. **c** Mouse RIPK3-flag reconstituted RIPK3-KO L929 cells were pretreated Quizartinib (2 μM) or vehicle for 1 h following treatment with TSZ for indicated time. Then cells were harvested and immunoprecipitated with M2 (anti-flag) antibody. The total cell lysates (TCL) and the immunoprecipitates were immunoblotted with the indicated antibodies. **d** L929 cells were pretreated Quizartinib (2 μM) or vehicle for 1 h following treatment with TSZ for indicated time. Then the cells were lysed with Triton X-100 lysis buffer. The insoluble fractions were collected and analyzed with the indicated antibodies. **e** L929 cells were pretreated vehicle or indicated compounds for 1 h following treatment with TSZ. Then the cell viabilities were determined using CCK8 method. *n* = 3 biologically independent samples. **f** Plasmid expressing HA-RIPK1 or Vector was transfected into 293T cells. Then the cells were treatment with indicated concentrations of Quizartinib, NEC-1 (10 μM) or vehicle for 20 h. The cells were harvested and analyzed with indicated antibodies. Data shown are representative of three independent experiments. Means ± SDs. ^#^*p* < 0.01.

To identify highly effective necroptosis inhibitors at low concentrations, we tested the ability of these 48 necroptosis inhibitors to inhibit TNF-induced necroptosis. As shown in Supplementary Fig. 10a, several compounds effectively interfered with TNF-induced necroptosis at 1 μM. Among these compounds, we noticed that Quizartinib inhibited TNF-induced necroptosis with higher efficacy than NEC-1 (Supplementary Fig. 10b). Importantly, Quizartinib showed limited toxicity towards cells (Supplementary Fig. 10c). Quizartinib also inhibited TSZ-induced necroptosis in HT-29 cells (Fig. 5a). Then, we investigated the mechanism by which Quizartinib inhibited TNF-induced necroptosis. Treatment with Quizartinib abolished TNF-induced phosphorylation of RIPK1, RIPK3 and MLKL in HT-29 cells (Fig. 5b), which is consistent with that in L929 cells (Supplementary Fig. 1b). Moreover, Quizartinib inhibited the interaction of RIPK1 and RIPK3 and the translocation of RIPK1, RIPK3 and MLKL into the Triton X-100 insoluble fraction (Fig. 5c, d). Since Quizartinib is a specific oral inhibitor of fms-related receptor tyrosine kinase 3 (FLT3), we investigated whether FLT3 is involved in TNF-induced necroptosis. As shown in Fig. 5e and Supplementary Fig. 11, the different FLT3 inhibitors gilteritinib, HM43239 and FLT3-IN-3 did not influence TNF-induced necroptosis, which indicated that inhibition of necroptosis by Quizartinib is independent of kinase activity of FLT3. Although Quizartinib inhibited the phosphorylation of RIPK1 induced by TNF (Supplementary Fig. 1b and Fig. 5b), Quizartinib, in contrast to NEC-1, did not inhibit the necroptosis induced by dimerization/oligomerization of RIPK1 (Supplementary Data 3). Moreover, NEC-1, but not Quizartinib, inhibited the phosphorylation of RIPK1 when RIPK1 was overexpressed (Fig. 5f). Furthermore, Quizartinib did not inhibit RIPK1 kinase activity in vitro (Supplementary Fig. 12a). The Cellular thermal shift assay (CETSA), which based on the principle that thermal stability of protein changes upon binding to the compound, was used to investigate the interaction of compound and its potential target protein^46^. As shown in Supplementary Fig. 12b, we found that Quizartinib did not influence the protein stability of RIPK1. Therefore, we proposed that Quizartinib did not regulate RIPK1 directly. Then, we investigated whether Quizartinib influences TNFR1 complex signaling and found that Quizartinib did not influence the phosphorylation of IκB or JNK induced by TNF (Supplementary Fig. 13a). Phosphorylation of RIPK1 at residue S321 by MAPK-activated protein kinase 2 (MK2) inhibited RIPK1 kinase activity^47–49^. Then, we tested whether Quizartinib regulated the phosphorylation of RIPK1 at residue S321. As shown in Supplementary Fig. 13b, Quizartinib did not interfere with the phosphorylation of RIPK1 at residue S321. Taken together, Quizartinib effectively inhibited TNF-induced necroptosis.

### Quizartinib protected mice from TNF-induced SIRS

**Fig. 6:**
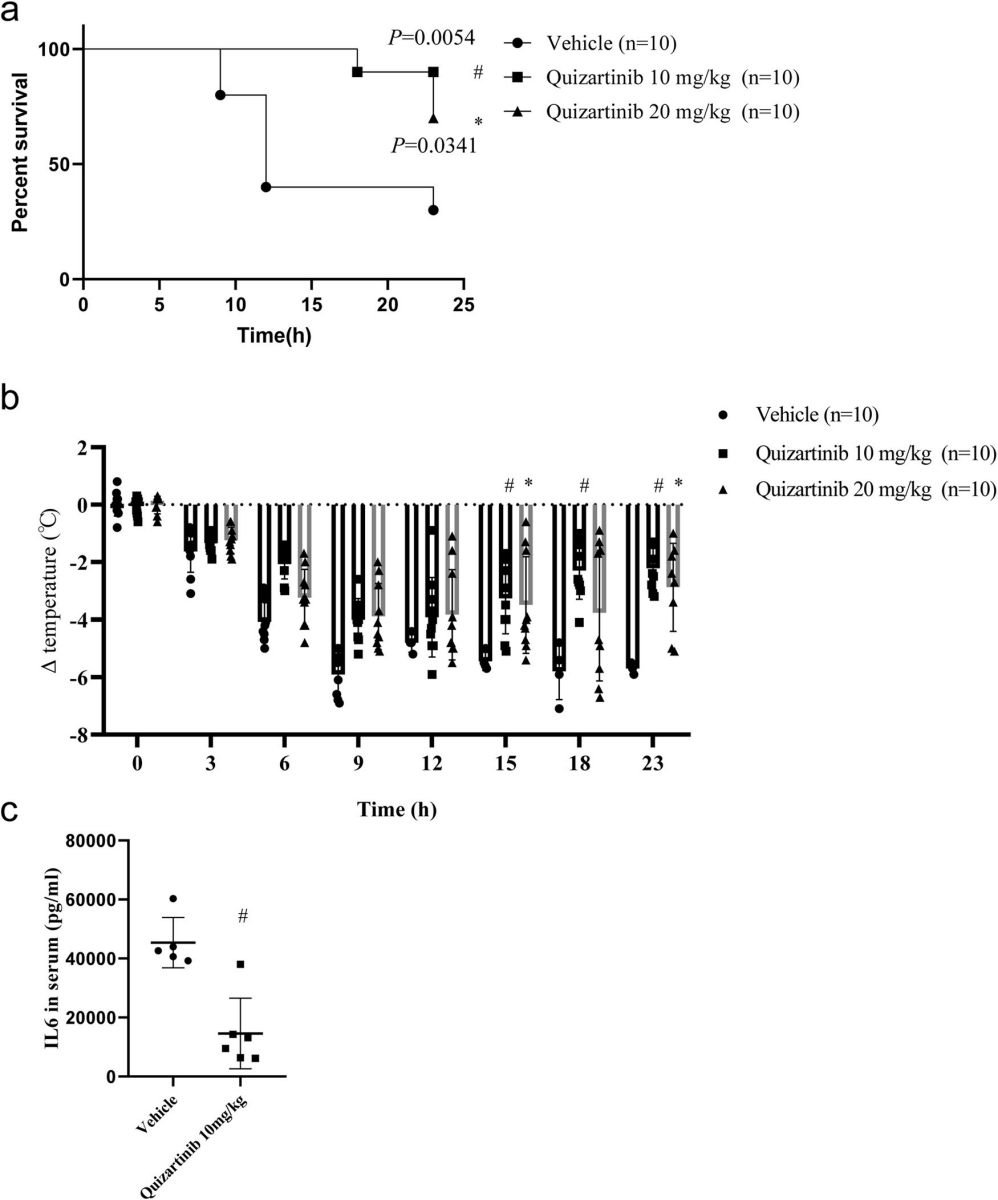
Quizartinib protected mice from TNF-induced SIRS. Mice were pretreated with vehicle or Quizartinib (10 mg/kg or 20 mg/kg gavage) for 1 h following injection with mTNF (10 μg/mouse i.v.). The survival curve (**a**) and body temperature loss (**b**) were determined. *n* = 10/group. **c** Plasma samples of mice treated with vehicle (*n* = 5, the beginning number of vehicle group was 6, however one mouse died before we collected plasma samples of mice) or Quizartinib (10 mg/kg gavage, *n* = 6) were collected 6 h after mTNF (10 μg/mouse i.v.) challenged. Serum levels of IL-6 were determined by ELISA. Data shown are representative of three independent experiments. Means ± SD. *p < 0.05, ^#^*p* < 0.01.

Necroptosis has been implicated in many pathological models, including the TNF-induced SIRS model. Then we tested whether Ibrutinib or Quizartinib were able to protects against TNF-induced SIRS. As shown in Supplementary Fig. 14, Ibrutinib (10 mg/kg, gavage) failed to protected mice from TNF-induced SIRS. In contrast, Quizartinib (10 mg/kg or 20 mg/kg, gavage) significantly protected mice from TNF-induced hypothermia and death (Fig. 6a, b and Supplementary Fig. 15a, b). Moreover, Quizartinib significantly reduced the serum interleukin-6 (IL-6) concentration induced by TNF (Fig. 6c and Supplementary Fig. 15c, d). Taken together, these data show that Quizartinib attenuated TNF-induced SIRS, which indicated that Quizartinib has potential clinical applications in necroptosis-associated diseases.

## Discussion

We performed necroptosis screening using a small-compound library and discovered dozens of compounds, including some FDA-approved/clinical trial drugs, that inhibited TNF-induced necroptosis. According to our analysis, these compounds seem to inhibit TNF-induced necroptosis through different mechanisms. Among these compounds, we found that Ibrutinib inhibited TNF-induced necroptosis by directly targeting RIPK3, while Quizartinib inhibited TNF-induced necroptosis by indirectly inhibiting the activation of RIPK1. Importantly, Quizartinib significantly ameliorated TNF-induced SIRS, which might have been due to its high efficiency in inhibiting necroptosis. Our study expands our knowledge of necroptosis inhibitors and provides potential therapeutic opportunities to treat necroptosis-related diseases using these FDA-approved/clinical trial drugs.

Ibrutinib is an oral inhibitor of Bruton tyrosine kinase (BTK) and has been approved by the FDA for the treatment of lymphoma. Ibrutinib has also been reported to inhibit TEC family and EGFR family kinases^50^. We noticed that Ibrutinib was reported to influence RIPK3 kinase activity in cardiac tissue using Chemoproteomic Kinase Profiling, but the detail mechanism was not investigated^51^. Here, we found that Ibrutinib inhibited TNF-induced RIPK3 autophosphorylation and recruitment and the activation of the downstream effector MLKL by RIPK3, thereby blocking TNF-induced necroptosis. Furthermore, our data indicated that Ibrutinib directly inhibited RIPK3 kinase activity, indicating that RIPK3 is a target of Ibrutinib. According to molecular docking simulation, Ibrutinib binds to the residues Leu183 and Val233 of RIPK3. The binding modes between Ibrutinib and GSK’872 with RIPK3 were different (Fig. 4d and Supplementary Fig. 6b). Interestingly, Thr182, which is nearby Leu183, was important for regulating RIPK3 kinase activity^52^. In contrast to Quizartinib that inhibited necroptosis even at 0.1 µM, Ibrutinib protected against TNF-induced necroptosis at concentrations ranging from 8 to 32 µM. Furthermore, high concentrations of Ibrutinib showed toxicity towards cells. These might account for why Ibrutinib failed to protected mice from TNF-induced SIRS. Ibrutinib formed a covalent bond with Cys-481 of BTK through its acrylamide warhead^43^. Interestingly, the acrylamide warhead of Ibrutinib, which was important for the interaction between Ibrutinib and BTK, was not required in its interaction with RIPK3 and the inhibition of TNF-induced necroptosis (Fig. 4d and Supplementary Fig. 8b). The syntheses of analog compounds based on the Ibrutinib scaffold will lead to the development of candidates that better inhibit necroptosis.

Quizartinib is a specific oral FLT3 inhibitor that has shown benefits for patients with FLT3-ITD acute myeloid leukemia (AML) in several clinical trials^53^. Our study indicated that Quizartinib inhibited TNF-induced necroptosis independent of FLT3. Quizartinib strongly inhibited the phosphorylation of RIPK1, RIPK3, and MLKL. Importantly, Quizartinib efficiently blocked necroptosis at low concentrations and protected mice from TNF-induced SIRS. We noticed that Quizartinib had been reported to bind to RIPK1 in a study of the interaction of kinases and kinase inhibitors^54^. However, Quizartinib, in contrast to NEC-1, neither protected against necroptosis induced by the dimerization/oligomerization of RIPK1 nor inhibited autophosphorylation of RIPK1 when RIPK1 was overexpressed. Moreover, Quizartinib was unable to inhibit full length RIPK1 kinase activity in vitro and did not increase protein stability of RIPK1 using CETSA method. Therefore, we propose that Quizartinib indirectly inhibits the autophosphorylation of RIPK1 induced by TNF. Identifying the target of Quizartinib will be of great interest and will expand our knowledge of the TNF-induced necroptosis signaling pathway.

Many necroptosis inhibitors had been uncovered by screening different compound libraries in different necroptosis models^27–29^. Among the necroptosis inhibitors identified in our screening, we found that some inhibitors are FDA-approved drugs. To determine whether these FDA-approved drugs can be repurposed for the treatment of necroptosis-related diseases, further extensive in vivo studies are needed. Moreover, we noticed that many necroptosis inhibitors identified in our screening were antitumour drugs/compounds. Considering that necroptosis has been reported to be involved in both tumor suppression and promotion^55,56^, whether inhibition of necroptosis by these antitumour drugs/compounds is contributes or counteracts cancer therapy remains to be studied. In conclusion, we identified dozens of necroptosis inhibitors and clarified the potential clinical applications of FDA-approved/clinical trial drugs to the treatment of necroptosis-related diseases.

## Methods

### Cell culture

The mouse fibrosarcoma L929 cell line, HEK293T cell line, NIH3T3-RIPK3 cell line, Hela-RIPK3 cell line and Mouse RIPK3-flag reconstituted RIPK3-KO L929 cell line were kindly provided by Professor Jiahuai Han (Xiamen University, Xiamen). L929, HEK293T, NIH3T3-RIPK3, Hela-RIPK3 and RIPK3-flag reconstituted RIPK3-KO L929 cells were maintained in Dulbecco’s Modified Eagle’s Medium (DMEM). The human colon cancer HT-29 cell line was maintained in RPMI-1640 medium. All of cell lines were supplemented with 10% fetal bovine serum, 4 mM L-glutamine, 100 IU penicillin and 100 mg/mL streptomycin at 37 °C in a humidified incubator containing 5% CO_2_. All of the cell lines have been authenticated using STR profiling.

### Reagents and antibodies

Small-compound library was purchased from APExBIO (USA). Pan-caspase inhibitor z-VAD, Necrostatin-1, GSK’872, Smac mimetic (SM-164), Ibrutinib, Quizartinib, BIIB068, RN486, Gilteritinib, HM43239, FLT3-IN-3, propidium iodide were purchased from TargetMol. 4-Hydroxytamoxifen (4-OHT) was purchased from medchemexpress (MCE) (USA). Cell Counting Kit-8 (CCK8) was purchased from cellcook (China). Recombined human RIPK1 and RIPK3 protein, MBP protein were purchased from signalchem (Canada). Recombinant Human TNF alpha was purchased from novoprotein (China). Recombinant mouse TNF alpha was purchased from Sino Biological (China). ADP-Glo Kinase Assay kit was purchased from promega (USA). Universal antibody diluent was purchased from NCM biotech (China). Rabbit anti-HA (51064-2-AP, 1:2000) and anti-Flag (20543-1-ap, 1:2000), mouse anti-GAPDH (60004-1-Ig, 1:2000), goat anti-mouse (SA00001-1, 1:10,000), and goat anti-Rat (SA00001-15, 1:10,000) antibodies were purchased from Proteintech (USA). Goat anti rabbit (A5014, 1:10,000) antibodies were purchased from ABclonal (USA). Rabbit anti JNK (9252T, 1:1000), p-JNK (T183/Y185) (4668T, 1:1000), p-IκB (2859T, 1:1000), p-mRIPK1 (S166) (53286S, 1:1000), p-mRIPK1 (S321) (38662S, 1:1000), hRIPK1 (3493T, 1:1000), hRIPK3 (13526S, 1:1000), hMLKL (14993S, 1:1000), p-hRIPK1 (S166) (65746S, 1:1000), p-hRIPK3 (S227) (93654S, 1:1000), p-hMLKL(S358) (91689S, 1:1000), and mouse anti-IκB (4814T, 1:1000) were purchased from Cell Signaling Technology (USA). Rabbit anti Vimentin (ab92547, 1:2000), p-mRIPK3 (S232/T231) (ab222320, 1:1000), p-mMLKL (S345) (ab196436, 1:1000), and rat anti-mMLKL (ab243142, 1:1000) were purchased from Abcam (England). Mouse anti-mRIPK1 (610458, 1:1000) was purchased from BD Biosciences (USA).

### Compound screen and cell viability assay

L929 cells were seeded into 96-well plates and pretreated with individual compound from small compound library for 1 h following treatment with TSZ for 4 h. Then the cell viability was determined using CCK8 method and absorbance was recorded with MULTISKAN GO microplate reader (Thermo scientific). For the primary screen (Fig. 1a, b), the absorbance of cells treated with individual compound and TSZ was normalized to the absorbance of cell treated with vehicle and TSZ (control). For other cell viability assay using CCK8 method, the absorbance of cells treated with vehicle was set to 100%. For FACS, cells were trypsinized, harvested and resuspended in PBS containing propidium iodide (PI). The proportions of PI-negative cells were quantified with FACSCanto (TM) II (BD). The specific gating strategies are listed in Supplementary Fig. 16.

### Artificially induced dimerization/oligomerization of RIPK1-HBD and RIPK3-HBD

Plasmids expressed RIPK1-HBD or RIPK3-HBD were kindly provided by prof. Jiahuai Han (Xiamen University, Xiamen)^32^. Artificially induced dimerization/oligomerization of RIPK1-HBD and RIPK3-HBD were induced by 4-hydroxytamoxifen (4-OHT)^57^. To induced dimerization/oligomerization of RIPK1-HBD or RIPK3-HBD mediated necroptosis, L929 cells stably expressing RIPK1-HBD or RIPK3-HBD were treated 4-OHT + z-VAD. The RIPK1 kinase inhibitor Nec-1 and RIPK3 kinase inhibitor GSK’872 were chose as control compound to interfere dimerization/oligomerization of RIPK1-HBD and RIPK3-HBD mediated necroptosis.

### Immunoprecipitation

Mouse RIPK3-flag reconstituted RIPK3-KO L929 was treated with compounds and TSZ for indicated times. Then the cells were harvested and lysed in cold lysis buffer (20 mM Tris-HCl pH 7.5, 120 mM NaCl, 1 mM EDTA, 1 mM EGTA, 1% Triton X-100, 2.5 mM Sodium pyrophosphate, 1 mM β-Glycerophosphate, 1 mM Na_3_VO_4_, 1 mM PMSF, Protease inhibitor cocktail (TargetMol)). After centrifugation at 20,000 × *g* for 30 min at 4 °C, the soluble fractions of cell lysates were collected and incubation with anti-flag M2 beads overnight. Then the beads were washed with cold lysis buffer three times. Each sample was added proper volume of sample buffer and performed western blotting analysis.

### In vitro kinase assay

The recombinant human RIPK1 and RIPK3 protein was incubated with the control DMSO or the Ibrutinib for 20 mins in the kinase assay buffer (40 mM tris-HCL, pH7.4, 20 mM MgCl_2_, 2.5 mM MnCl_2_, 0.1 mg/ml BSA, 50 μM DTT). Then the substrate MBP and ATP were added to the reaction at room temperature for 1.5 h. The kinase activity was determined using ADP-Glo Kinase Assay kit method (Promega).

### Docking simulations

Molecular Docking simulations were performed with the software of AutoDock Vina^58^. The crystal structure of the published RIPK3 (PDB code: 7MX3)^38^ was retrieved from the RCSB Protein Data Bank. The solvent molecules and original ligand within the protein structure were removed in the docking calculations, and then the ligands were docked to the protein structure to calculate the dock score.

### Cellular thermal shift assay (CETSA)

L929 cells were incubated with Quizartinib (2 μM) or DMSO for 3 h. The cells were harvested and incubated at different temperatures (from 37 °C to 55 °C) for 3 min. Then the cells were freeze-thawed for three times, and centrifuged at 12,000 rpm for 20 min at 4 °C. The cell lysates were mixed with loading buffer and denatured at 95 °C for 10 min. The samples were analyzed using Western blotting.

### TNF-induced systemic inflammatory response syndrome

C57BL/6 male mice (8–12 weeks old) were purchased from GemPharmatech Co.,Ltd. (Nanjing, China) and bred under standard conditions. All animal experiments were performed in accordance with protocols approved by Institutional Animal Ethical Committee of Fujian Medical University. Ibrutinib and Quizartinib were dissolved with DMSO and mixed with corn oil as a ratio of 1:9. Mice were gavage with or without Quizartinib (10 mg/kg or 20 mg/kg) or Ibrutinib (10 mg/kg) for 1 h followed by injecting intravenously with mouse TNF alpha (10 μg/mouse). Mortality and temperature of mice were monitored every 3 h. Plasma samples of indicated mice were collected 6 h after mTNF challenged. Serum levels of IL-6 were determined by ELISA.

### Statistics and reproducibility

All of data were analyzed using GraphPad Prism 8 software. Results are represented as means ± SD. Two-tailed Student’s *t* test was used to compare differences between two groups. The log-rank (Mantel–Cox) test was performed for mice survival curve analysis. Statistical significance was defined as *p < 0.05, ^#^*p* < 0.01.

### Reporting summary

Further information on research design is available in the Nature Portfolio Reporting Summary linked to this article.

### Supplementary information

Supplementary Figures

Description of Additional Supplementary Files

Supplementary Data 1

Supplementary Data 2

Supplementary Data 3

Supplementary Data 4

Reporting Summary

## Data Availability

All source data for the graphs and charts in the main and Supplementary Figures are present in Supplementary Data 4. Uncropped gel images are included in Supplementary Fig. 17. Additional data are available from the corresponding author on reasonable request.

## References

[1] Han J, Zhong CQ, Zhang DW (2011). Programmed necrosis: backup to and competitor with apoptosis in the immune system. Nat. Immunol..

[2] Chan FK, Luz NF, Moriwaki K (2015). Programmed necrosis in the cross talk of cell death and inflammation. Annu. Rev. Immunol..

[3] Zhang DW (2009). RIP3, an energy metabolism regulator that switches TNF-induced cell death from apoptosis to necrosis. Science.

[4] He S (2009). Receptor interacting protein kinase-3 determines cellular necrotic response to TNF-alpha. Cell.

[5] Cho YS (2009). Phosphorylation-driven assembly of the RIP1-RIP3 complex regulates programmed necrosis and virus-induced inflammation. Cell.

[6] Chen X (2022). Mosaic composition of RIP1-RIP3 signalling hub and its role in regulating cell death. Nat. Cell Biol..

[7] Mompean M (2018). The structure of the necrosome RIPK1-RIPK3 core, a human hetero-amyloid signaling complex. Cell.

[8] Grootjans S, Vanden Berghe T, Vandenabeele P (2017). Initiation and execution mechanisms of necroptosis: an overview. Cell Death Differ..

[9] Sun L (2012). Mixed lineage kinase domain-like protein mediates necrosis signaling downstream of RIP3 kinase. Cell.

[10] Zhao J (2012). Mixed lineage kinase domain-like is a key receptor interacting protein 3 downstream component of TNF-induced necrosis. Proc. Natl Acad. Sci. USA.

[11] Murphy JM (2013). The pseudokinase MLKL mediates necroptosis via a molecular switch mechanism. Immunity.

[12] Xie T (2013). Structural insights into RIP3-mediated necroptotic signaling. Cell Rep..

[13] Cai Z (2014). Plasma membrane translocation of trimerized MLKL protein is required for TNF-induced necroptosis. Nat. Cell Biol..

[14] Wang H (2014). Mixed lineage kinase domain-like protein MLKL causes necrotic membrane disruption upon phosphorylation by RIP3. Mol. Cell.

[15] Chen X (2014). Translocation of mixed lineage kinase domain-like protein to plasma membrane leads to necrotic cell death. Cell Res..

[16] Zhang Y (2021). The MLKL kinase-like domain dimerization is an indispensable step of mammalian MLKL activation in necroptosis signaling. Cell Death Dis..

[17] McNamara DE (2019). Direct activation of human MLKL by a select repertoire of inositol phosphate metabolites. Cell Chem. Biol..

[18] Galluzzi L, Kepp O, Chan FK, Kroemer G (2017). Necroptosis: mechanisms and relevance to disease. Annu. Rev. Pathol..

[19] Wang R (2020). Gut stem cell necroptosis by genome instability triggers bowel inflammation. Nature.

[20] Lin J (2013). A role of RIP3-mediated macrophage necrosis in atherosclerosis development. Cell Rep..

[21] Kaczmarek A, Vandenabeele P, Krysko DV (2013). Necroptosis: the release of damage-associated molecular patterns and its physiological relevance. Immunity.

[22] Degterev A (2005). Chemical inhibitor of nonapoptotic cell death with therapeutic potential for ischemic brain injury. Nat. Chem. Biol..

[23] Degterev A (2008). Identification of RIP1 kinase as a specific cellular target of necrostatins. Nat. Chem. Biol..

[24] Kaiser WJ (2013). Toll-like receptor 3-mediated necrosis via TRIF, RIP3, and MLKL. J. Biol. Chem..

[25] Mandal P (2014). RIP3 induces apoptosis independent of pronecrotic kinase activity. Mol. Cell.

[26] Zhuang C, Chen F (2020). Small-molecule inhibitors of necroptosis: current status and perspectives. J. Med. Chem..

[27] Martens S (2017). Sorafenib tosylate inhibits directly necrosome complex formation and protects in mouse models of inflammation and tissue injury. Cell Death Dis..

[28] Fauster A (2015). A cellular screen identifies ponatinib and pazopanib as inhibitors of necroptosis. Cell Death Dis..

[29] Li JX (2014). The B-Raf(V600E) inhibitor dabrafenib selectively inhibits RIP3 and alleviates acetaminophen-induced liver injury. Cell Death Dis..

[30] Li D (2015). A cytosolic heat shock protein 90 and cochaperone CDC37 complex is required for RIP3 activation during necroptosis. Proc. Natl Acad. Sci. USA.

[31] Wang Y (2021). Discovery of bardoxolone derivatives as novel orally active necroptosis inhibitors. Eur. J. Med. Chem..

[32] Zhang Y (2017). RIP1 autophosphorylation is promoted by mitochondrial ROS and is essential for RIP3 recruitment into necrosome. Nat. Commun..

[33] Cook WD (2014). RIPK1- and RIPK3-induced cell death mode is determined by target availability. Cell Death Differ..

[34] Orozco S (2014). RIPK1 both positively and negatively regulates RIPK3 oligomerization and necroptosis. Cell Death Differ..

[35] Wu XN (2014). Distinct roles of RIP1-RIP3 hetero- and RIP3-RIP3 homo-interaction in mediating necroptosis. Cell Death Differ..

[36] Yu L (2023). Repositioning linifanib as a potent anti-necroptosis agent for sepsis. Cell Death Discov..

[37] Pierotti CL (2023). The VEGFR/PDGFR tyrosine kinase inhibitor, ABT-869, blocks necroptosis by targeting RIPK1 kinase. Biochem. J..

[38] Meng Y (2021). Human RIPK3 maintains MLKL in an inactive conformation prior to cell death by necroptosis. Nat. Commun..

[39] Petrie EJ (2018). Conformational switching of the pseudokinase domain promotes human MLKL tetramerization and cell death by necroptosis. Nat. Commun..

[40] Meng Y (2022). Human RIPK3 C-lobe phosphorylation is essential for necroptotic signaling. Cell Death Dis..

[41] Chen W (2013). Diverse sequence determinants control human and mouse receptor interacting protein 3 (RIP3) and mixed lineage kinase domain-like (MLKL) interaction in necroptotic signaling. J. Biol. Chem..

[42] Amin P (2018). Regulation of a distinct activated RIPK1 intermediate bridging complex I and complex II in TNFalpha-mediated apoptosis. Proc. Natl Acad. Sci. USA.

[43] Honigberg LA (2010). The Bruton tyrosine kinase inhibitor PCI-32765 blocks B-cell activation and is efficacious in models of autoimmune disease and B-cell malignancy. Proc. Natl Acad. Sci. USA.

[44] Burger JA (2015). Ibrutinib as initial therapy for patients with chronic lymphocytic leukemia. N. Engl. J. Med..

[45] Sun X (1999). RIP3, a novel apoptosis-inducing kinase. J. Biol. Chem..

[46] Martinez Molina D (2013). Monitoring drug target engagement in cells and tissues using the cellular thermal shift assay. Science.

[47] Dondelinger Y (2017). MK2 phosphorylation of RIPK1 regulates TNF-mediated cell death. Nat. Cell Biol..

[48] Jaco I (2017). MK2 phosphorylates RIPK1 to prevent TNF-induced cell death. Mol. Cell.

[49] Menon MB (2017). p38(MAPK)/MK2-dependent phosphorylation controls cytotoxic RIPK1 signalling in inflammation and infection. Nat. Cell Biol..

[50] Wu J, Liu C, Tsui ST, Liu D (2016). Second-generation inhibitors of Bruton tyrosine kinase. J. Hematol. Oncol..

[51] Xiao L (2020). Ibrutinib-mediated atrial fibrillation attributable to inhibition of C-terminal Src kinase. Circulation.

[52] Choi SW (2018). PELI1 selectively targets kinase-active RIP3 for ubiquitylation-dependent proteasomal degradation. Mol. Cell.

[53] Zhou F, Ge Z, Chen B (2019). Quizartinib (AC220): a promising option for acute myeloid leukemia. Drug Des. Dev. Ther..

[54] Davis MI (2011). Comprehensive analysis of kinase inhibitor selectivity. Nat. Biotechnol..

[55] Yan J, Wan P, Choksi S, Liu ZG (2022). Necroptosis and tumor progression. Trends Cancer.

[56] Najafov A, Chen H, Yuan J (2017). Necroptosis and cancer. Trends Cancer.

[57] Gallinari P (2005). A functionally orthogonal estrogen receptor-based transcription switch specifically induced by a nonsteroid synthetic ligand. Chem. Biol..

[58] Trott O, Olson AJ (2010). AutoDock Vina: improving the speed and accuracy of docking with a new scoring function, efficient optimization, and multithreading. J. Comput. Chem..

