# Supplementary material for: Repurposing of Ibrutinib and Quizartinib as potent inhibitors of necroptosis
Source: Commun Biol. 2023 Sep 23;6:972. doi: 10.1038/s42003-023-05353-5 (PMC10517925; doi:10.1038/s42003-023-05353-5)
Supplement: Supplementary file 1 — Supplementary Figures [file 42003_2023_5353_MOESM1_ESM.pdf]

a

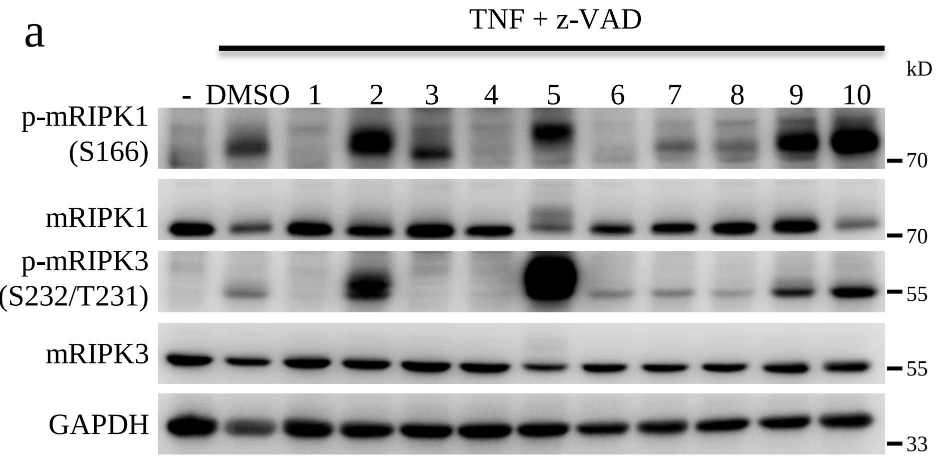

	Item Name
1	Linifanib (ABT-869)
2	Cabozantinib (XL184, BMS-907351)
3	PCI-32765 (Ibrutinib)
4	Sorafenib
5	(R)-Crizotinib
6	Pazopanib (GW-786034)
7	Afatinib dimaleate
8	Pelitinib (EKB-569)
9	Cediranib (AZD217)
10	Dasatinib (BMS-354825)

b

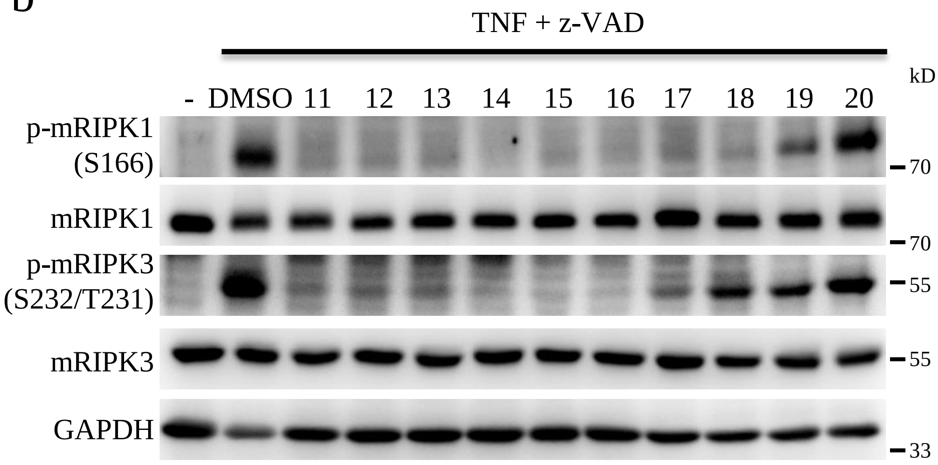

	Item Name
11	Nordihydroguaiaretic acid
12	Regorafenib hydrochloride
13	Regorafenib monohydrate
14	Quizartinib (AC220)
15	Regorafenib
16	Sorafenib Tosylate
17	Neratinib (HKI-272)
18	BIRB 796 (Doramapimod)
19	Afatinib (BIBW2992)
20	Dacomitinib (PF299804, PF299)

c

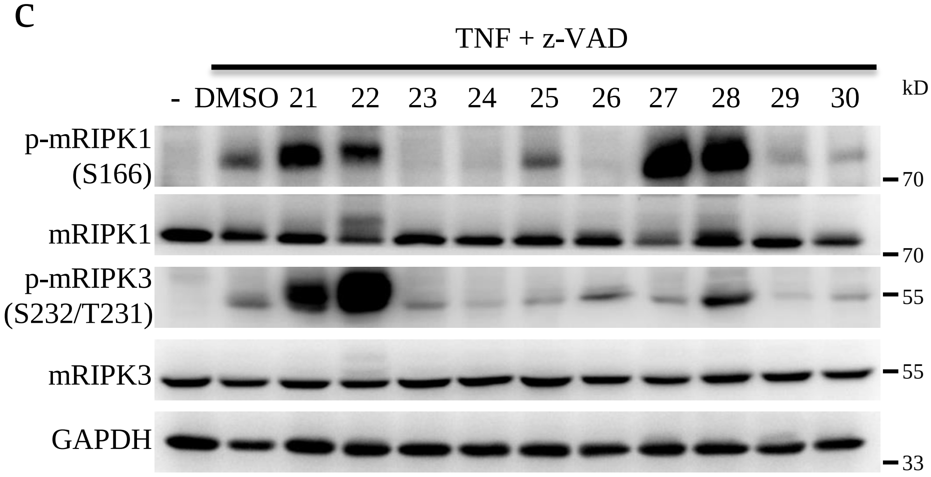

	Item Name
21	Cabozantinib malate (XL184)
22	Crizotinib hydrochloride
23	Pazopanib Hydrochloride
24	Axitinib (AG 013736)
25	CH5424802
26	Dovitinib Dilactic acid
27	Poziotinib
28	Dasatinib Monohydrate
29	Pexidartinib (PLX3397)
30	Entrectinib

d

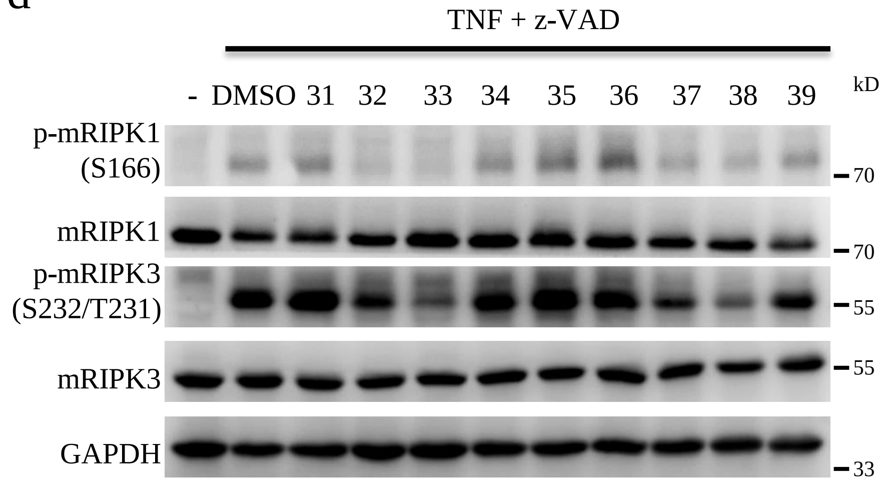

	Item Name
31	Bardoxolone methyl
32	AT13387
33	Brexpiprazole
34	Fenretinide
35	YM155
36	Menadione
37	Vortioxetine (Lu AA21004) HBr
38	Vortioxetine
39	Nitazoxanide

e

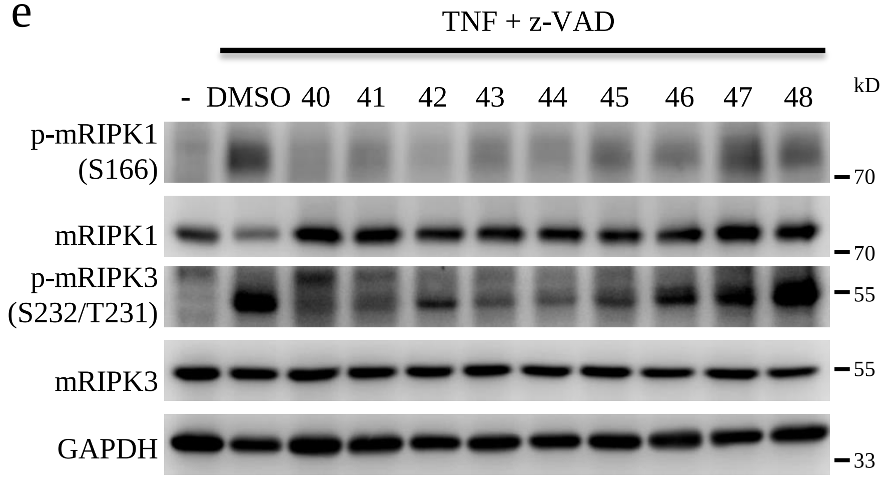

	Item Name
40	Primidone
41	Pyrocatechol
42	Aripiprazole
43	Paroxetine HCl
44	Sertraline HCl
45	Hydroquinone
46	Ethyl gallate
47	Parthenolide
48	Nitroxoline

Supplementary Figure 1. The influence of necroptosis inhibitors we identified on TNF-induced phosphorylation of RIPK1 and RIPK3. (a)-(e) L929 cells were pretreated with individual compound (15 μM) for 1 hour following stimulation with TNF (10 ng/ml) and pan-caspase inhibitor z-VAD (10 μM) (TZ) to induce necroptosis. Then the cells were harvested and analyzed with the indicated antibodies.

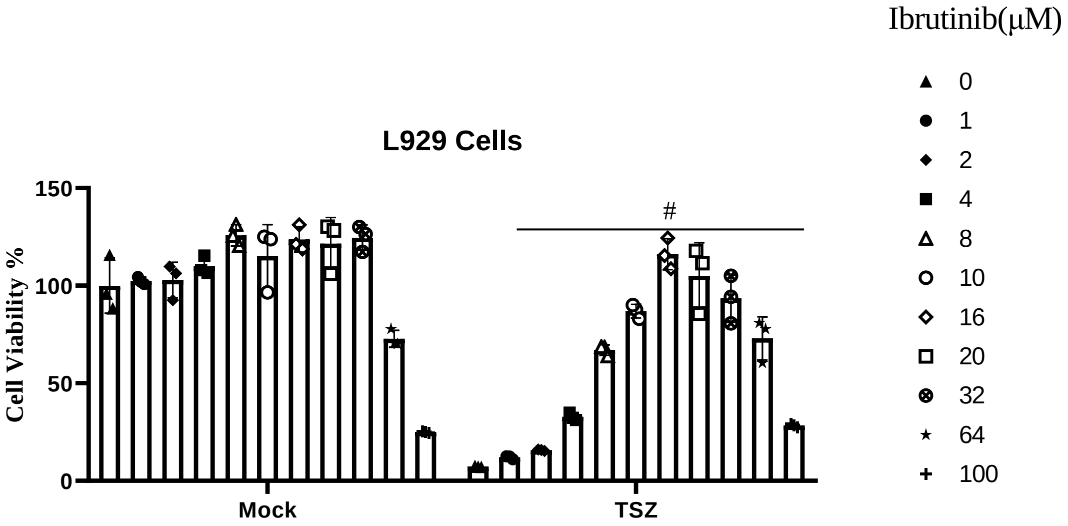

Supplementary Figure 2. Ibrutinib inhibited TSZ-induced necroptosis in an dose dependent manner. L929 cells were pretreated with different concentrations of Ibrutinib for 1 hour following stimulation with TNF (10 ng/ml), Smac mimetic (SM-164) (0.1 μM) and pan-caspase inhibitor z-VAD (10 μM) (TSZ) to induce necroptosis. Cell viabilities were determined with Cell Counting Kit-8 method. #*p*<0.01. n=3.

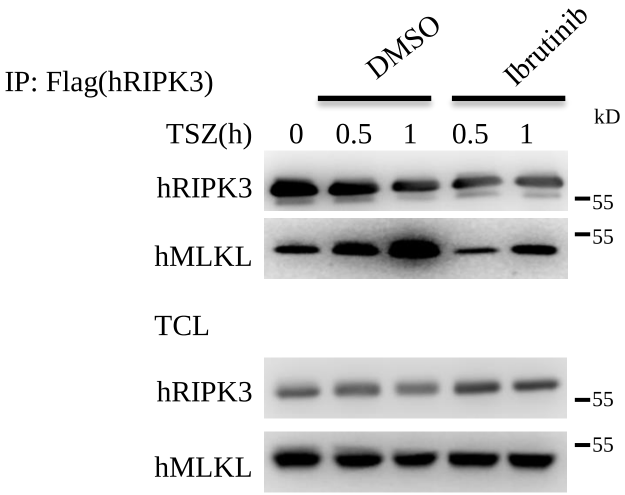

Supplementary Figure 3. Hela-RIPK3(Flag) were pretreated Ibrutinib (15 μM) or vehicle for 1 hour following treatment with TSZ for indicated time. Then cells were harvested and immunoprecipitated with M2 (anti-flag) antibody. The total cell lysates (TCL) and the immunoprecipitates were immunoblotted with the indicated antibodies.

a

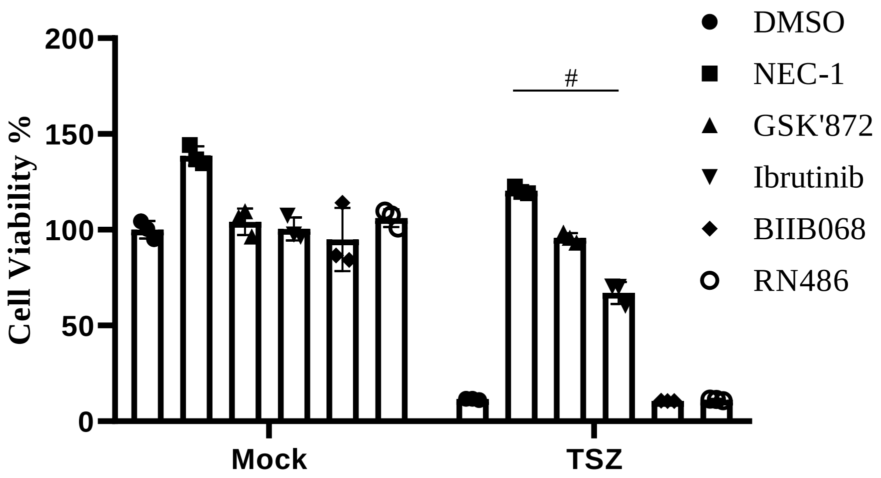

b

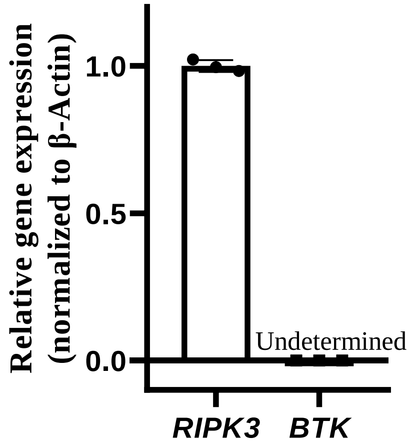

Supplementary Figure 4. BTK was not involved in TNF-induced necroptosis in L929 cells. (a) L929 cells were pretreated with NEC-1 (20 μM), GSK’872 (10 μM), Ibrutinib (10 μM), BTK inhibitor BIIB068 (10 μM), BTK inhibitor RN486 (10 μM) or vehicle for 1 hour following stimulation with TSZ. Cell viabilities were determined using CCK8 method. n=3. #*p*<0.01. (b) The expression levels of *RIPK3* and *BTK* were determined using realtime-PCR method in L929 cells. n=3.

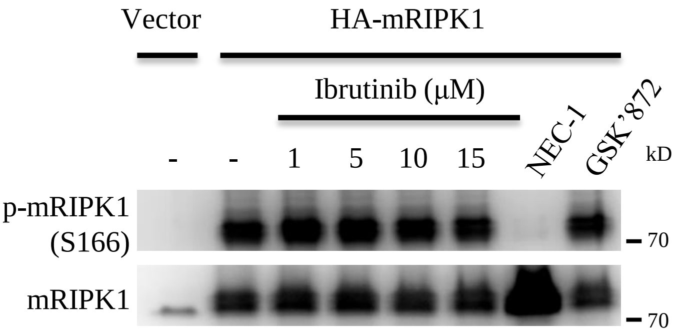

Supplementary Figure 5. Ibrutinib did not influence phosphorylation of RIPK1 when overexpressed. Plasmid expressing HA-mRIPK1 or Vector was transfected into 293T cells. Then the cells were stimulated with indicated concentrations of Ibrutinib, GSK'872 (10 μM), NEC-1 (10 μM) for 20 hours. The cells were harvested and analyzed with indicated antibodies.

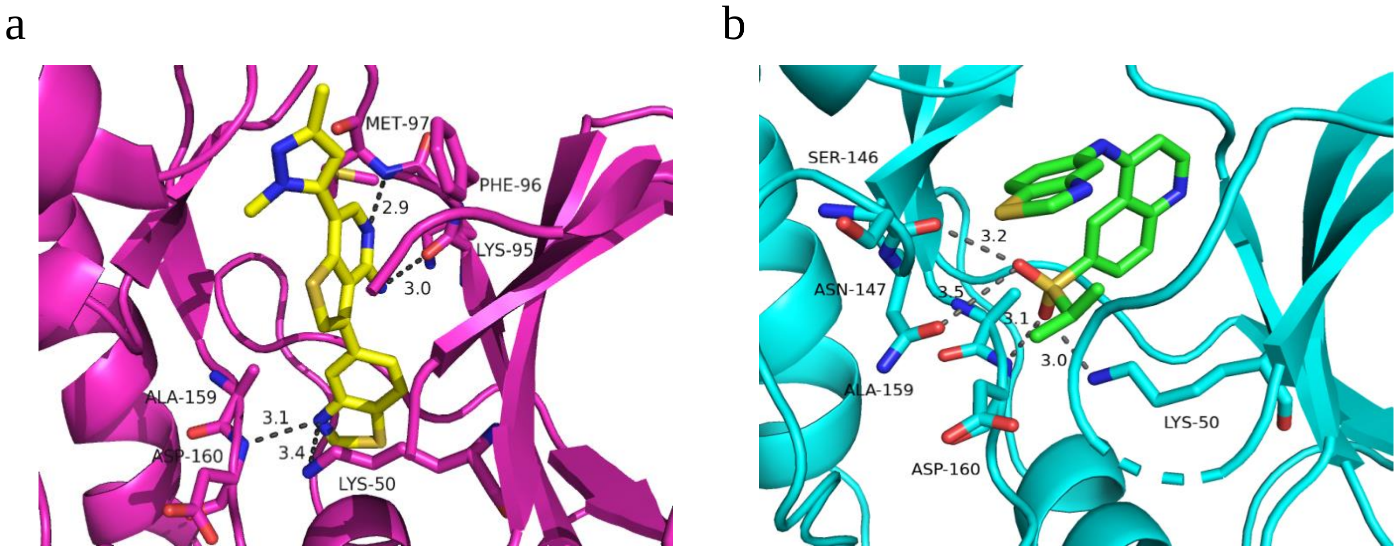

Supplementary Figure 6. The interaction between RIPK3 and small compounds. (a) The crystal structure of human RIPK3 complexed with specific RIPK3 inhibitor GSK’843 (PDB code: 7MX3) [38]. (b) Molecular Docking simulation result of GSK’872 and RIPK3 (PDB code: 7MX3) [38].

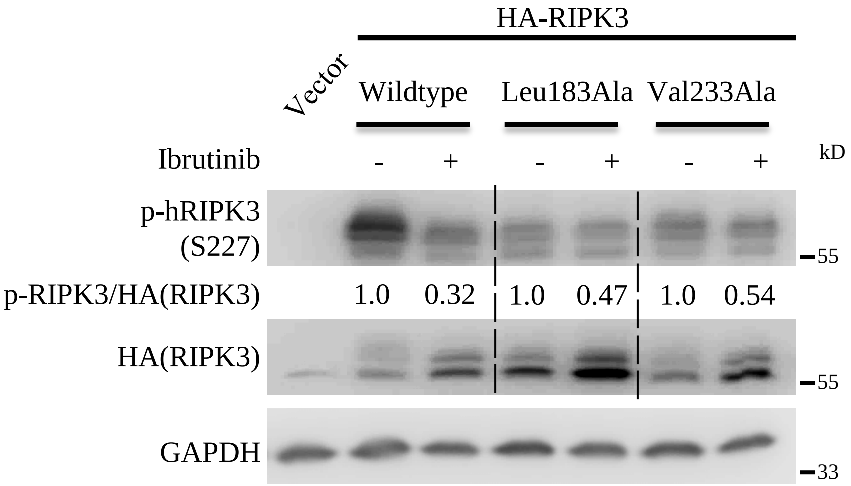

Supplementary Figure 7. Ibrutinib is less sufficient in inhibition of autophosphorylation level of Leu183Ala or Val233Ala RIPK3 when compared to wildtype RIPK3. Plasmid expressing HA-RIPK3 or Vector was transfected into 293T cells. Then the cells were stimulated with indicated concentrations of Ibrutinib (15 μM) for 14 hours. The cells were harvested and analyzed with indicated antibodies.

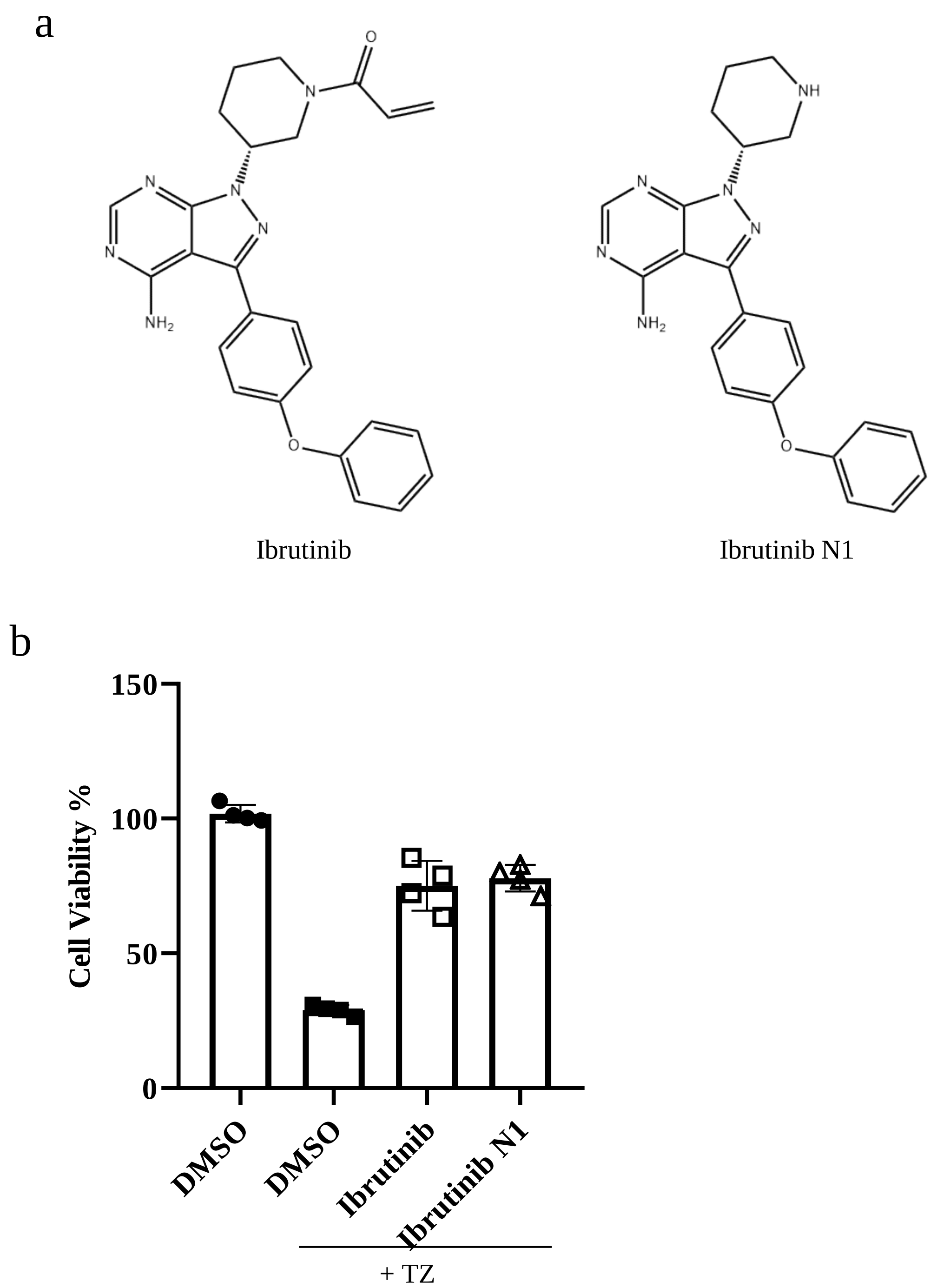

Supplementary Figure 8. Both Ibrutinib and its analogues Ibrutinib N1 were able to inhibited TNF-induced necroptosis. (a) The structure of Ibrutinib and Ibrutinib N1. (b) L929 cells were pretreated with Ibrutinib (8 μM), Ibrutinib N1 (8 μM) or vehicle for 1hour following treatment with TZ. Cell viabilities were determined using CCK8 method. n=4.

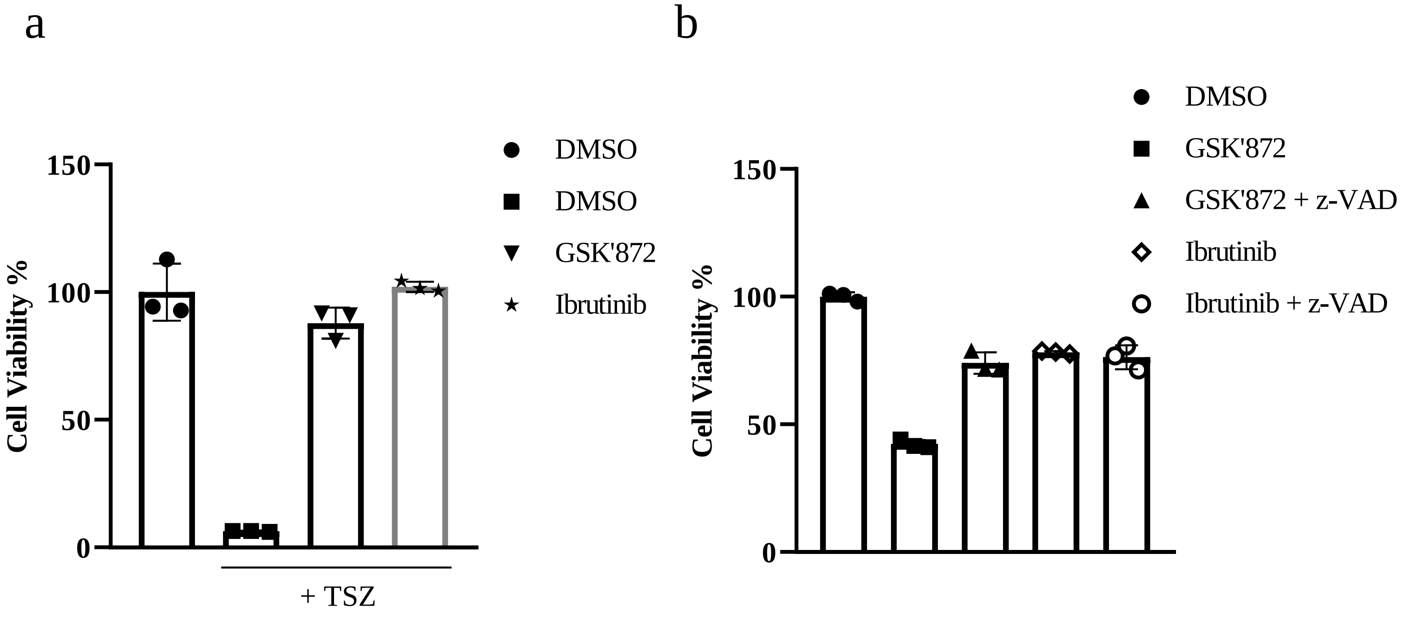

Supplementary Figure 9. Ibrutinib inhibited TNF-induced necroptosis and did not inducing apoptosis. (a) L929 cells were pretreated GSK'872 (10 μM), Ibrutinib (10 μM) or vehicle for 1hour following treatment with TSZ for 3 hours. Cell viabilities were determined using CCK8 method. n=3. (b) L929 cells were treated with GSK'872 (10 μM), Ibrutinib (10 μM) or vehicle for 10 hours, then cell viabilities were determined using CCK8 method. n=3.

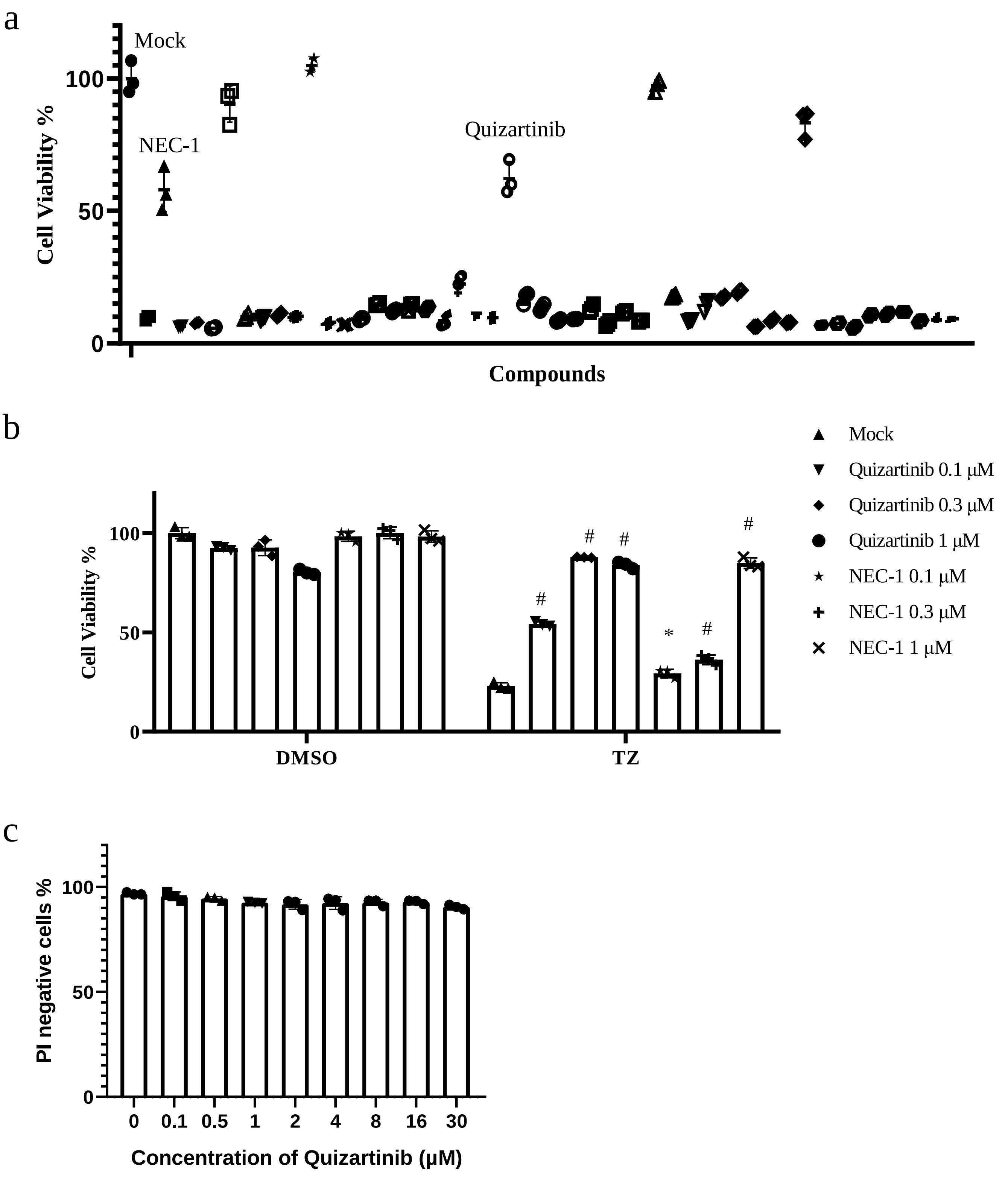

Supplementary Figure 10. Quizartinib effectively inhibited TNF-induced necroptosis. (a) L929 cells were pretreated with NEC-1 (1 μM) or necroptosis inhibitors (1 μM) we identified for 1 hour following stimulation with TSZ. Then the cell viabilities were determined using CCK8 method. n=3. (b) L929 cells were pretreated with indicated concentration of NEC-1 or Quizartinib for 1 hour following stimulation with TSZ. Then the cell viabilities were determined using CCK8 method. n=3. (c) L929 cells were treated with indicated concentration of Quizartinib for 24 hours. Then the cell viabilities were determined using propidium iodide (PI) exclusion. n=3. **p*<0.05, #*p*<0.01.

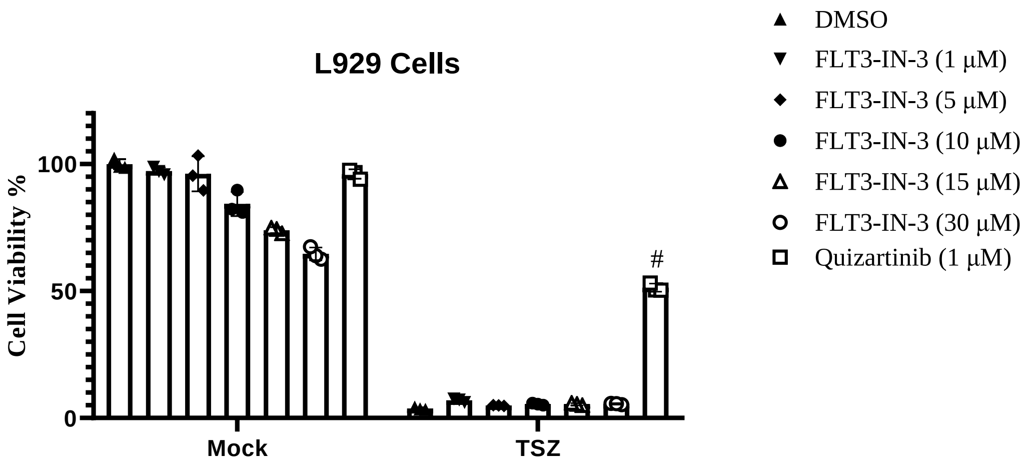

Supplementary Figure 11. Quizartinib, but not FLT3 inhibitor FLT3-IN-3, inhibited TNF-induced necroptosis. L929 cells were pretreated with different concentrations of FLT3-IN-3 or Quizartinib (1 μM) for 1 hour following stimulation with TSZ to induce necroptosis. Cell viabilities were determined with Cell Counting Kit-8 method. n=3. #*p*<0.01.

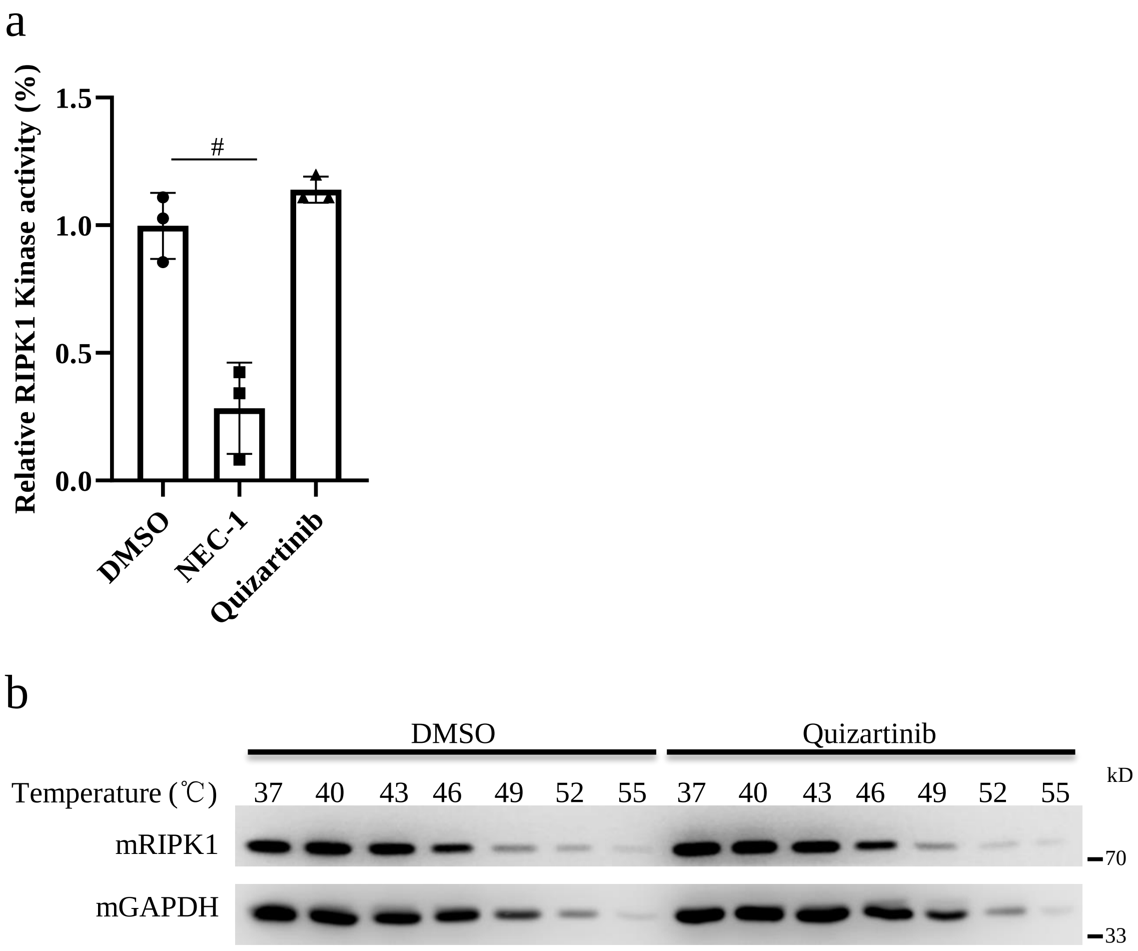

Supplementary Figure 12. Quizartinib did not target RIPK1 directly. (a) In vitro ADP-Glo kinase assay using recombinant hRIPK1 protein. Recombinant hRIPK1 was incubated with DMSO, NEC-1 (10 μM) or Quizartinib (10 μM). n=3. (b) The protein stability of RIPK1 were determent using CETSA assay method in L929 cells treated with DMSO or Quizartinib (2 μM).

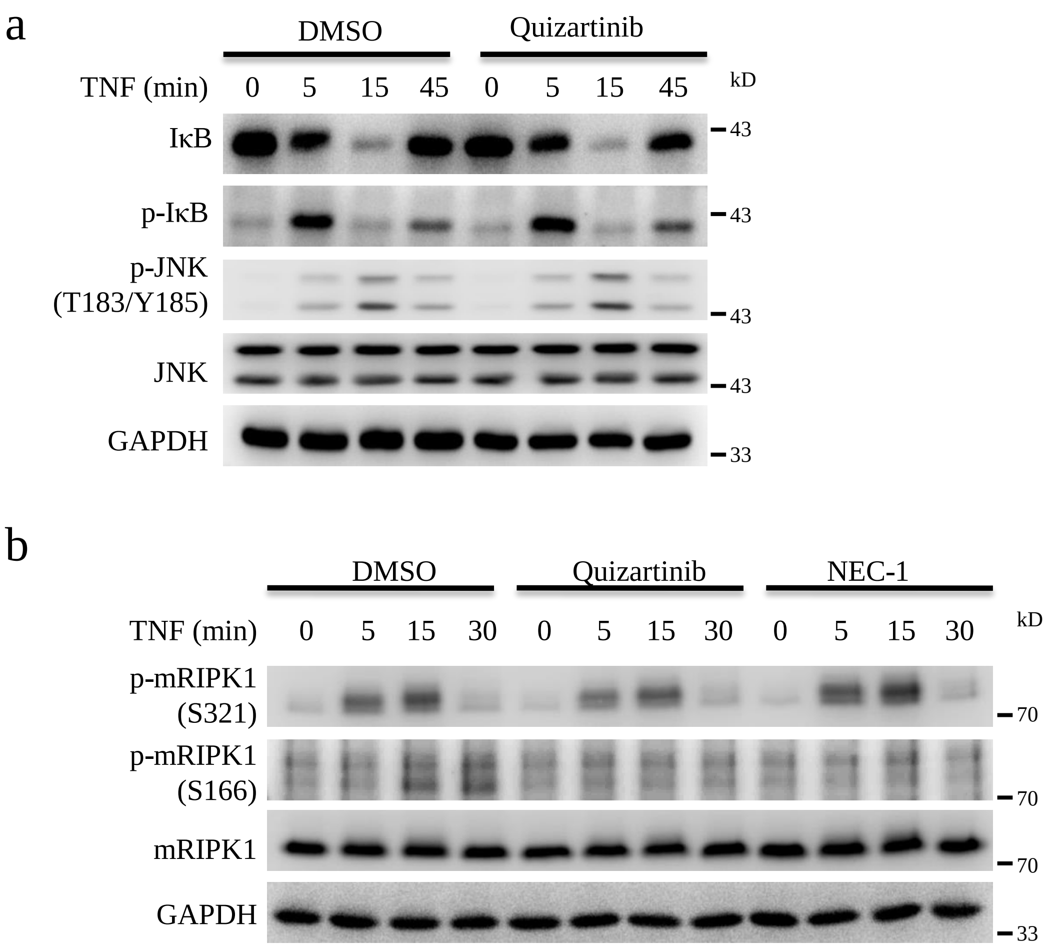

Supplementary Figure 13. Quizartinib did not influence TNFR1 mediated NF-κB and MAPK signaling and the S321 phosphorylation of RIPK1. (a) L929 cells were pretreated with or without Quizartinib (2 μM) for 1 hour following stimulation with TNF for indicated times. Then the cells were harvested and analyzed with the indicated antibodies. (b) L929 cells were pretreated with vehicle, Quizartinib (2 μM) or NEC-1 (10 μM) for 1 hour following stimulation with TNF for indicated times. Then the cells were harvested and analyzed with the indicated antibodies.

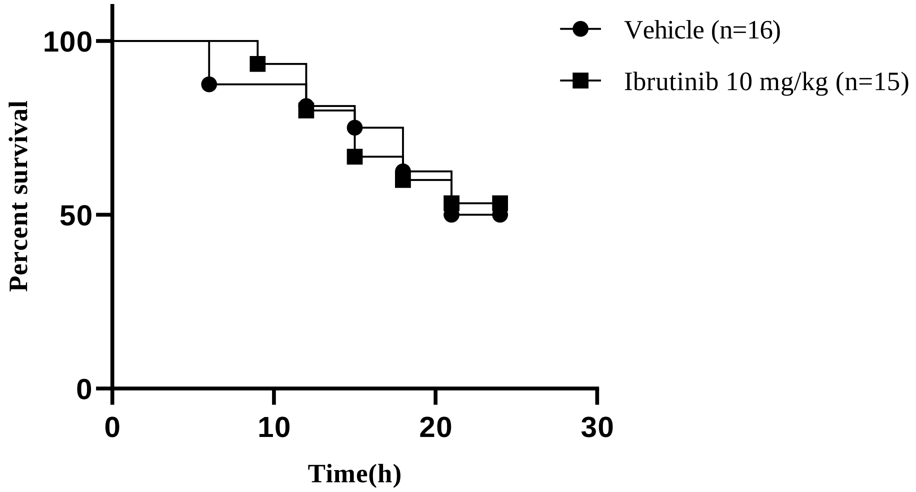

Supplementary Figure 14. Ibrutinib did not protected mice from TNF-induced SIRS. Mice were pretreated with vehicle or Ibrutinib (10 mg/kg gavage) for 1 hour following injection with mTNF (10 μg/mouse i.v.). The survival curve were determined.

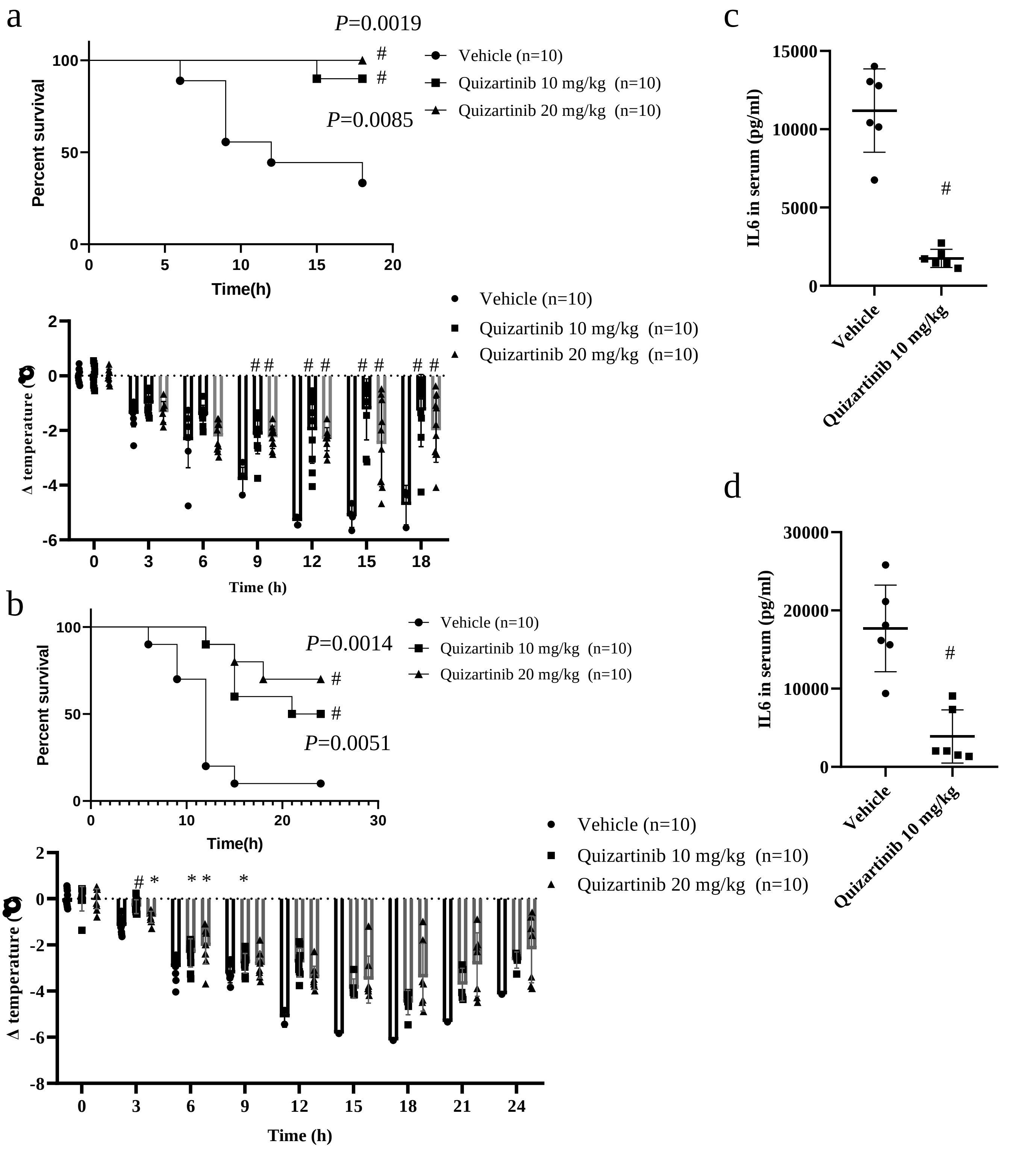

Supplementary Figure 15. Replicate experiments related to Figure 6. (a) and (b) Replicate experiments for Figure 6a and b. Mice were pretreated with vehicle or Quizartinib (10 mg/kg or 20 mg/kg gavage) for 1 hour following injection with mTNF (10 μg/mouse i.v.). The survival curve and body temperature loss were determined. n=10/group. For Fig. S14B, since only two mouse survival post 12 hours after injection with mTNF, the t-test for body temperature loss was not performed. (c) and (d) Replicate experiments for Figure 6c. Plasma samples of mice treated with vehicle (n=6) or Quizartinib (10 mg/kg gavage, n=6) were collected 6 hours after mTNF (10μg/mouse i.v.) challenged. Serum levels of IL-6 were determined by ELISA. Data shown are representative of three independent experiments. Means ± SD.*$p<0.05$, #$p<0.01$.

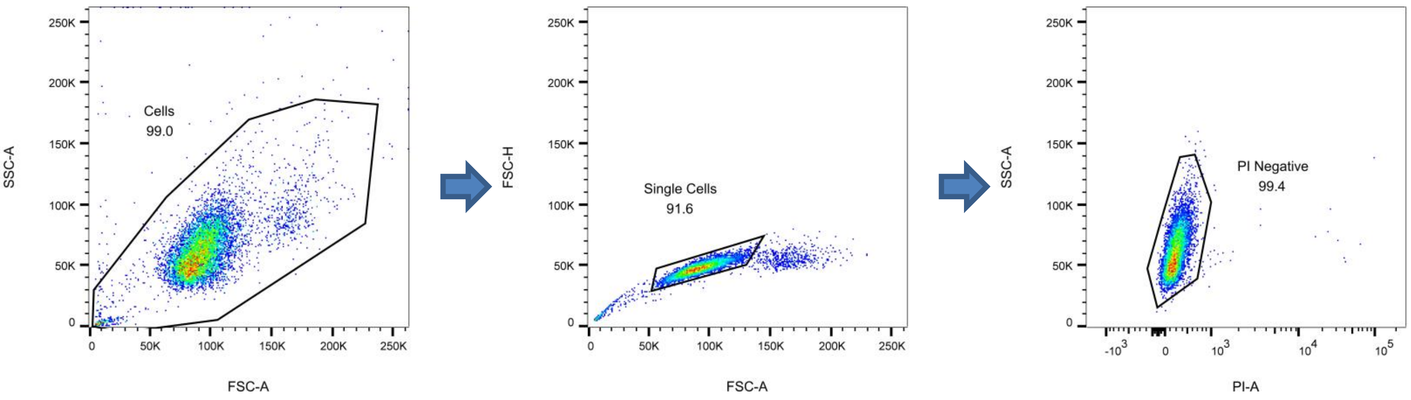

Supplementary Figure 16. Flow cytometry gating strategy for cell viability analysis. The cell gate was selected in a SSC-A versus FSC-A plot, followed by a gating in a FSC-H versus FSC-A plot. Cells were further plotted as SSC-A versus PI to identify survival or death cells.

Supplementary Figure 17. Uncropped gel images

Figure 3B

p-hRIPK1
(S166)

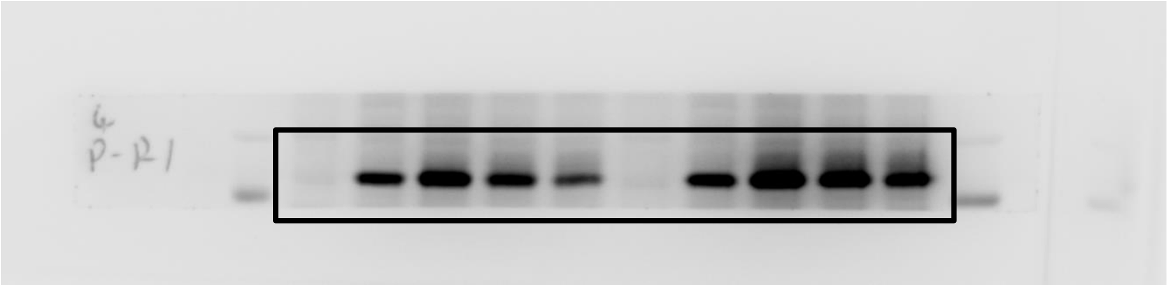

hRIPK1

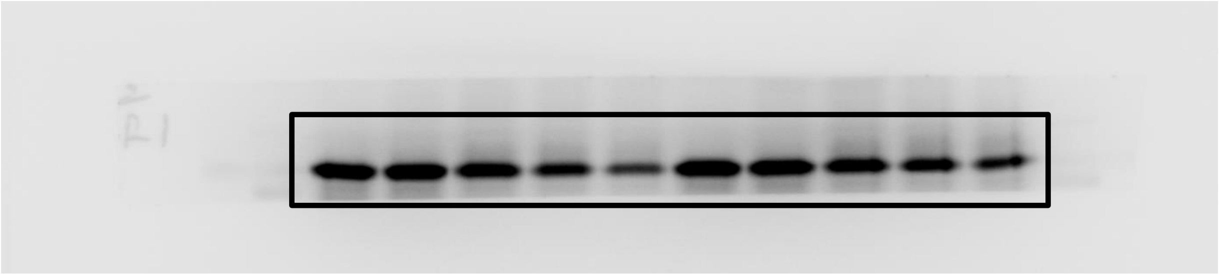

p-hRIPK3
(S227)

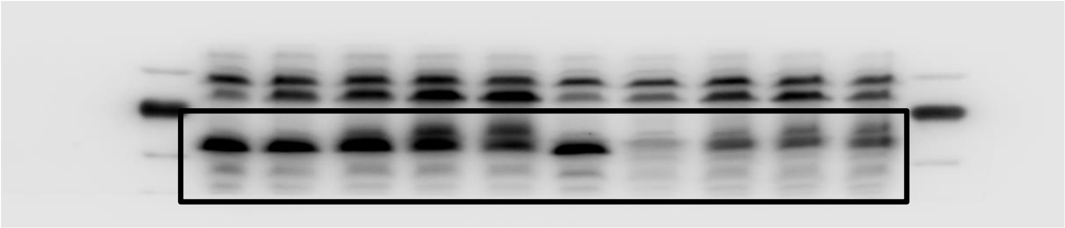

hRIPK3

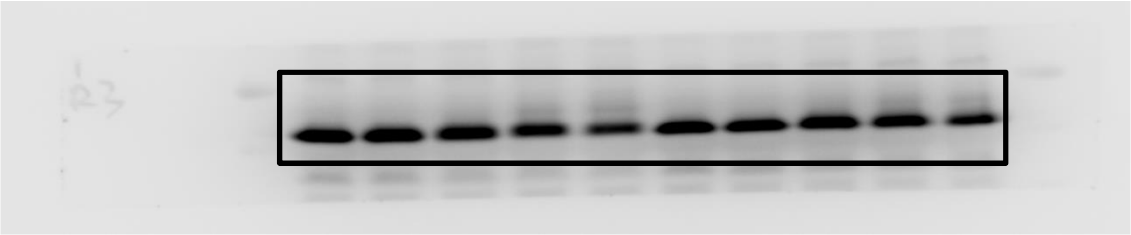

p-hMLKL
(S358)

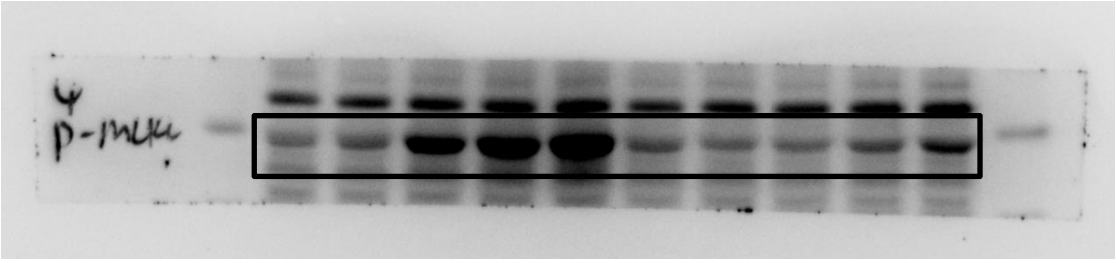

hMLKL

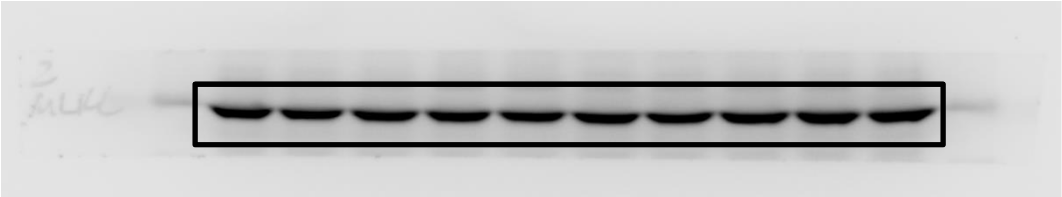

GAPDH

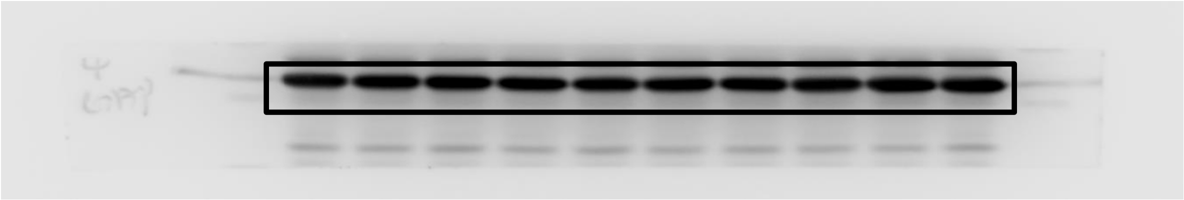

Figure 3C

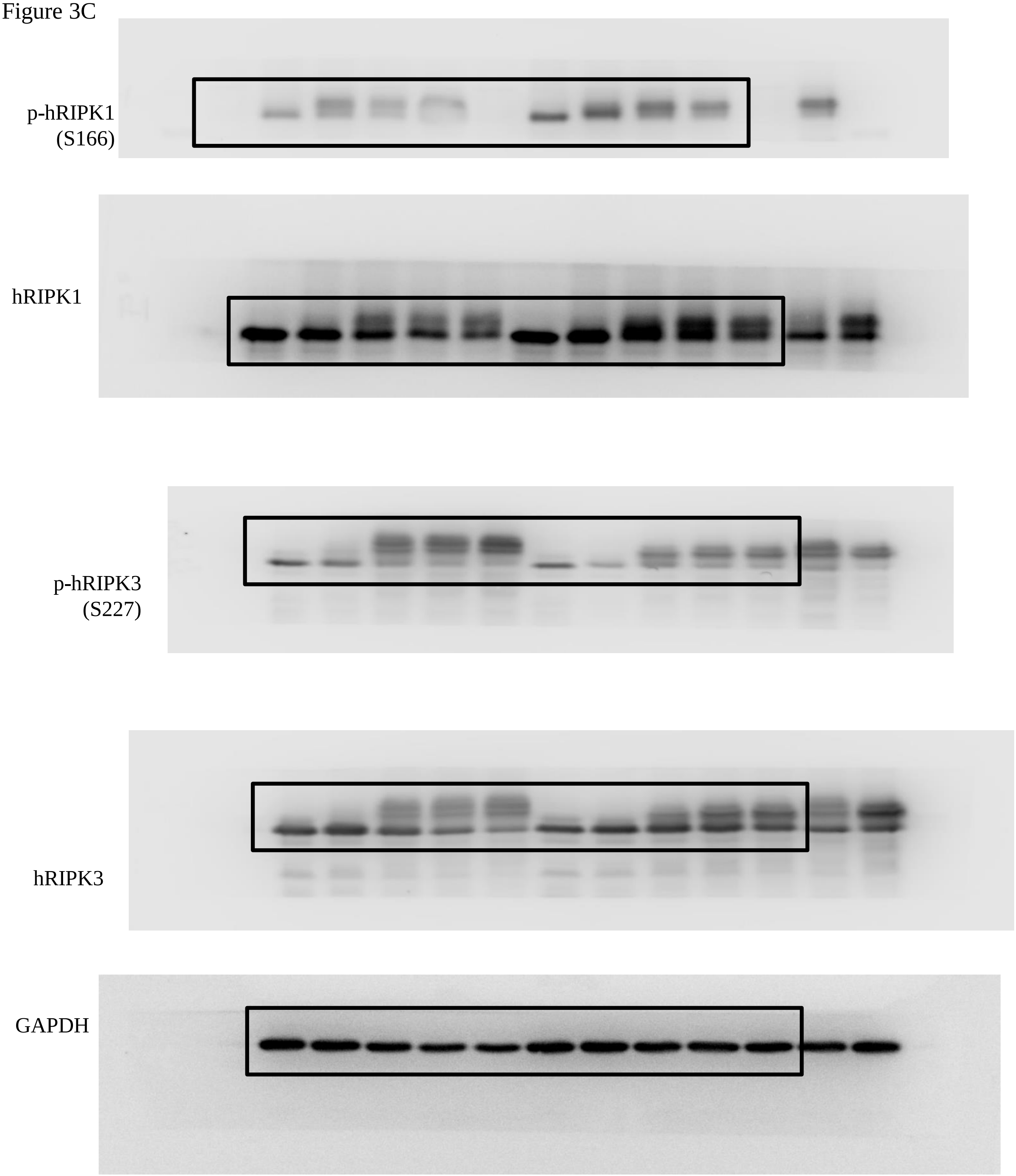

Figure 3D

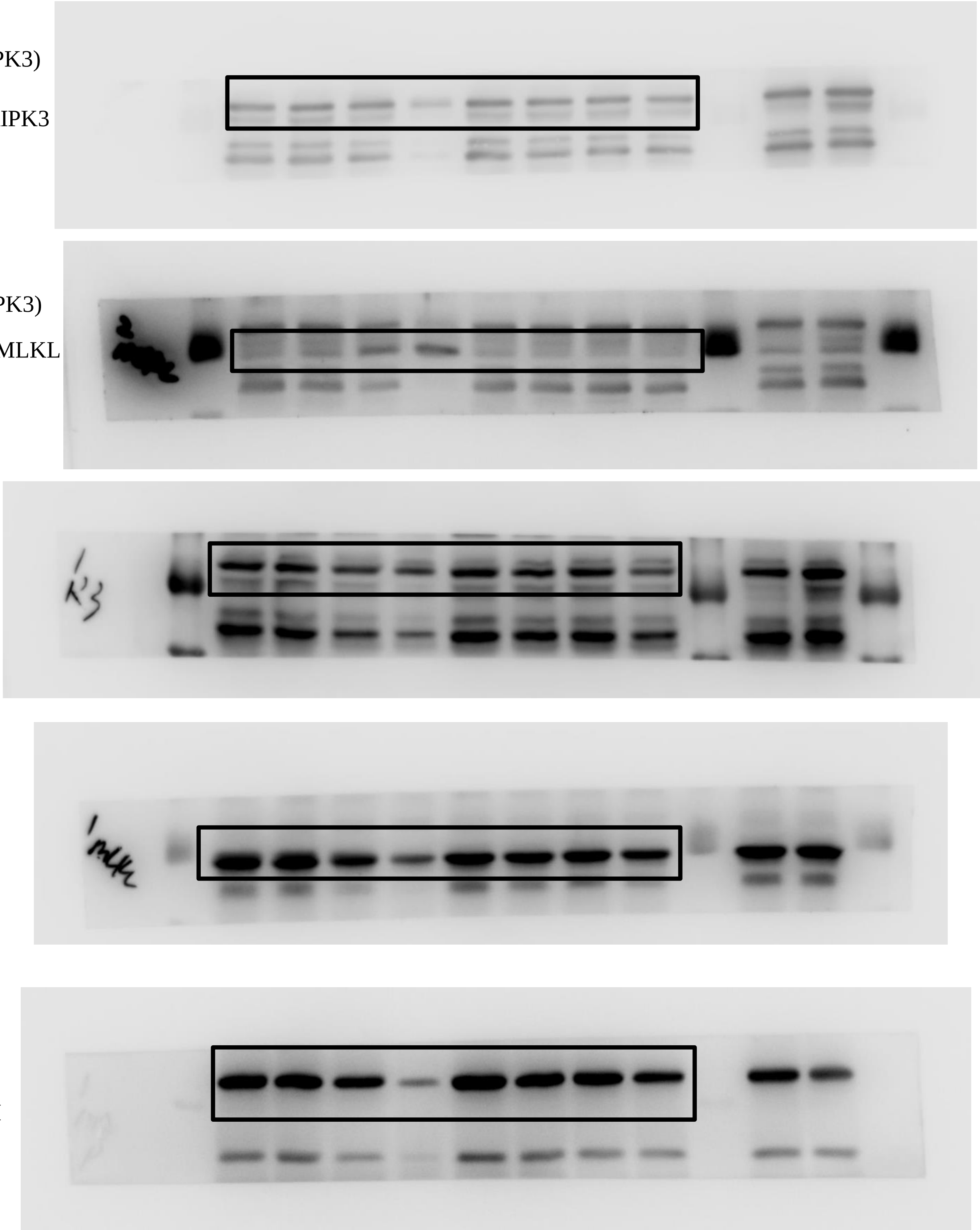
IP: Flag (mRIPK3)
mRIPK3
IP: Flag (mRIPK3)
mMLKL
TCL
mRIPK3
TCL
mMLKL
TCL
GAPDH

Figure 3E

mRIPK1

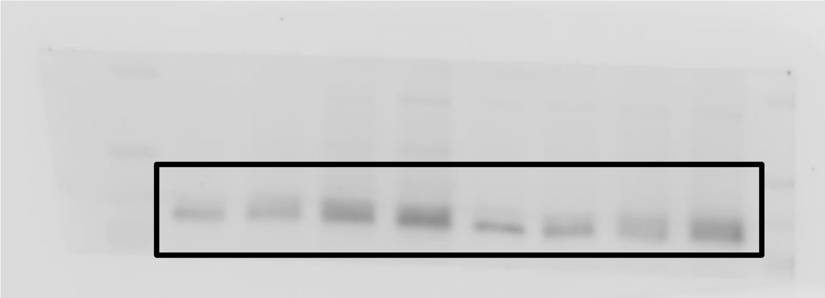

mRIPK3

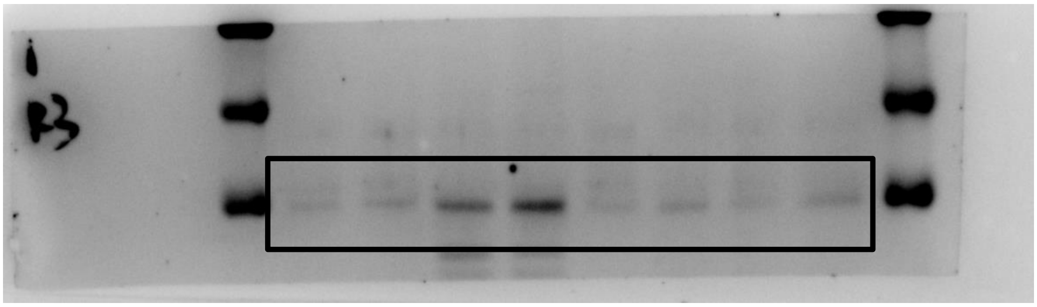

mMLKL

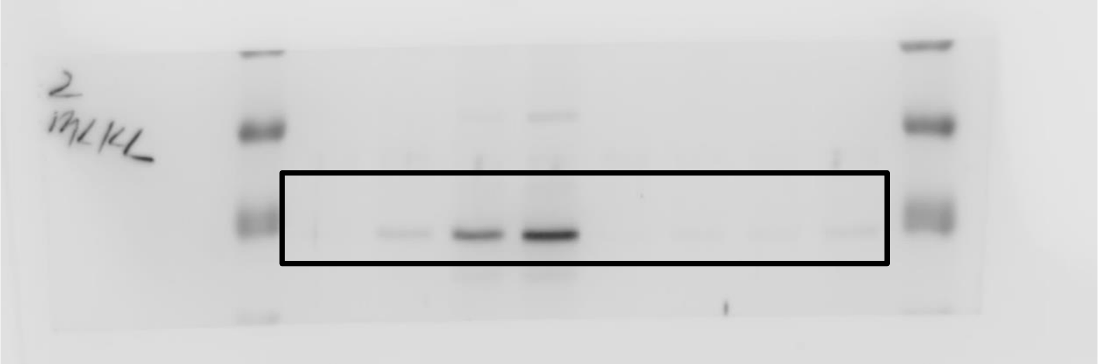

VIMENTIN

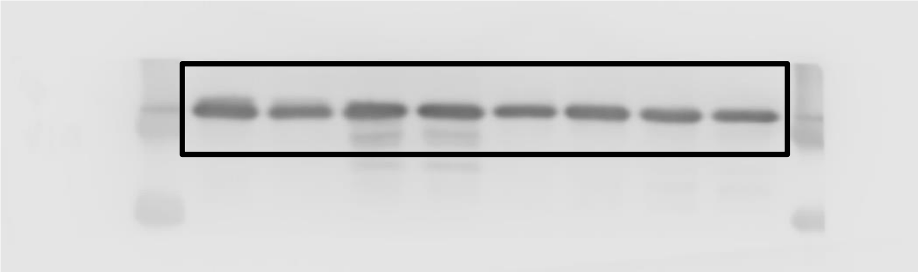

Figure 4A

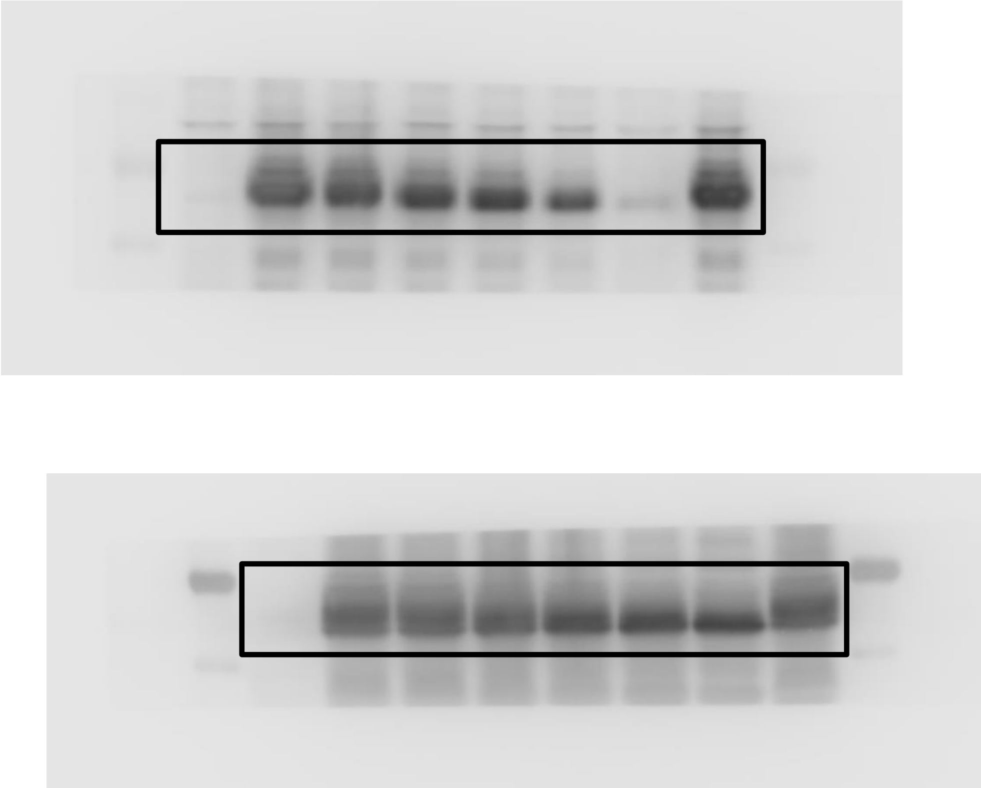

p-mRIPK3
(S232/T231)

HA

Figure 4B

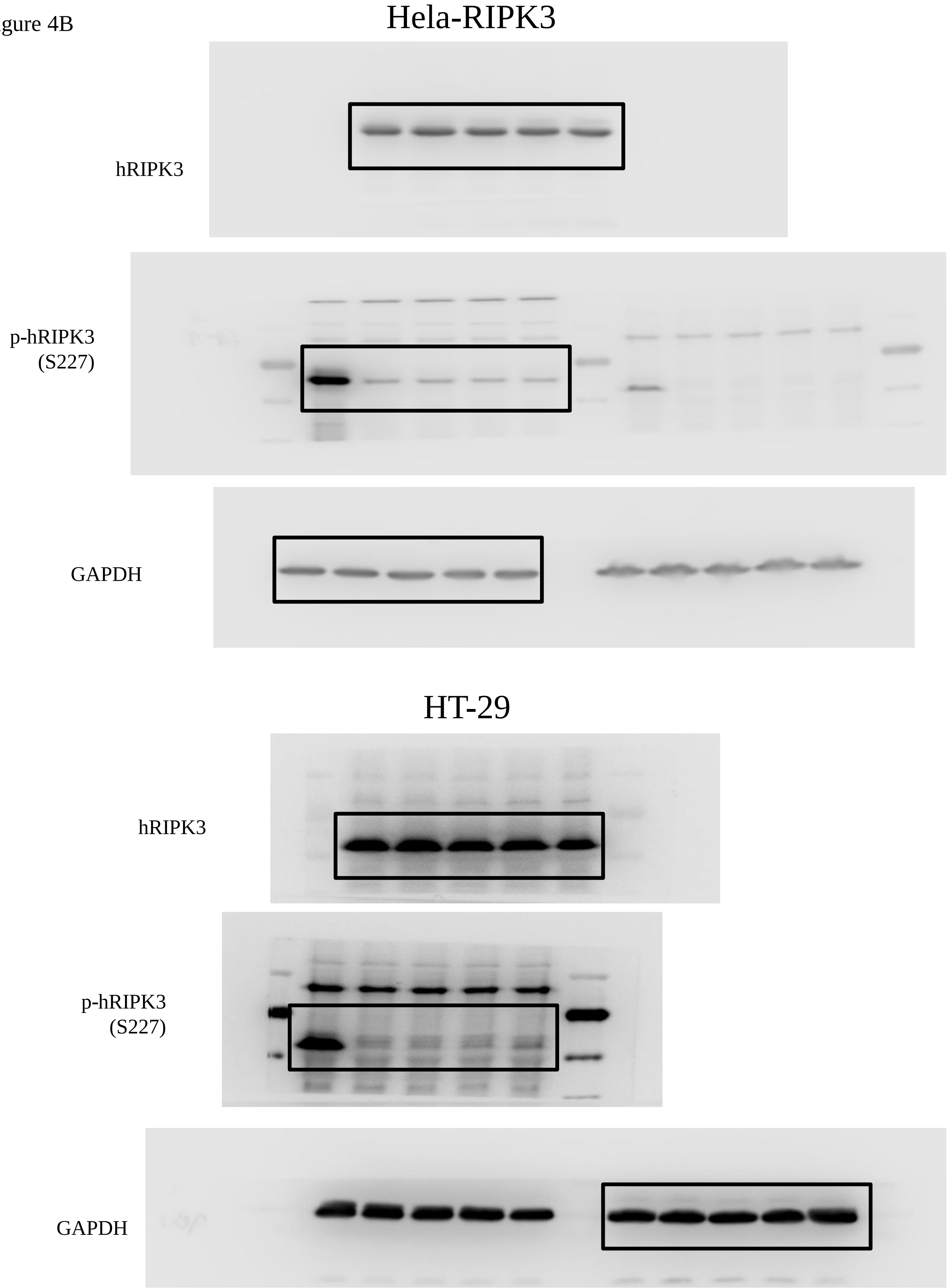
Hela-RIPK3
hRIPK3
p-hRIPK3
(S227)
GAPDH
HT-29
hRIPK3
p-hRIPK3
(S227)
GAPDH

Figure 4F

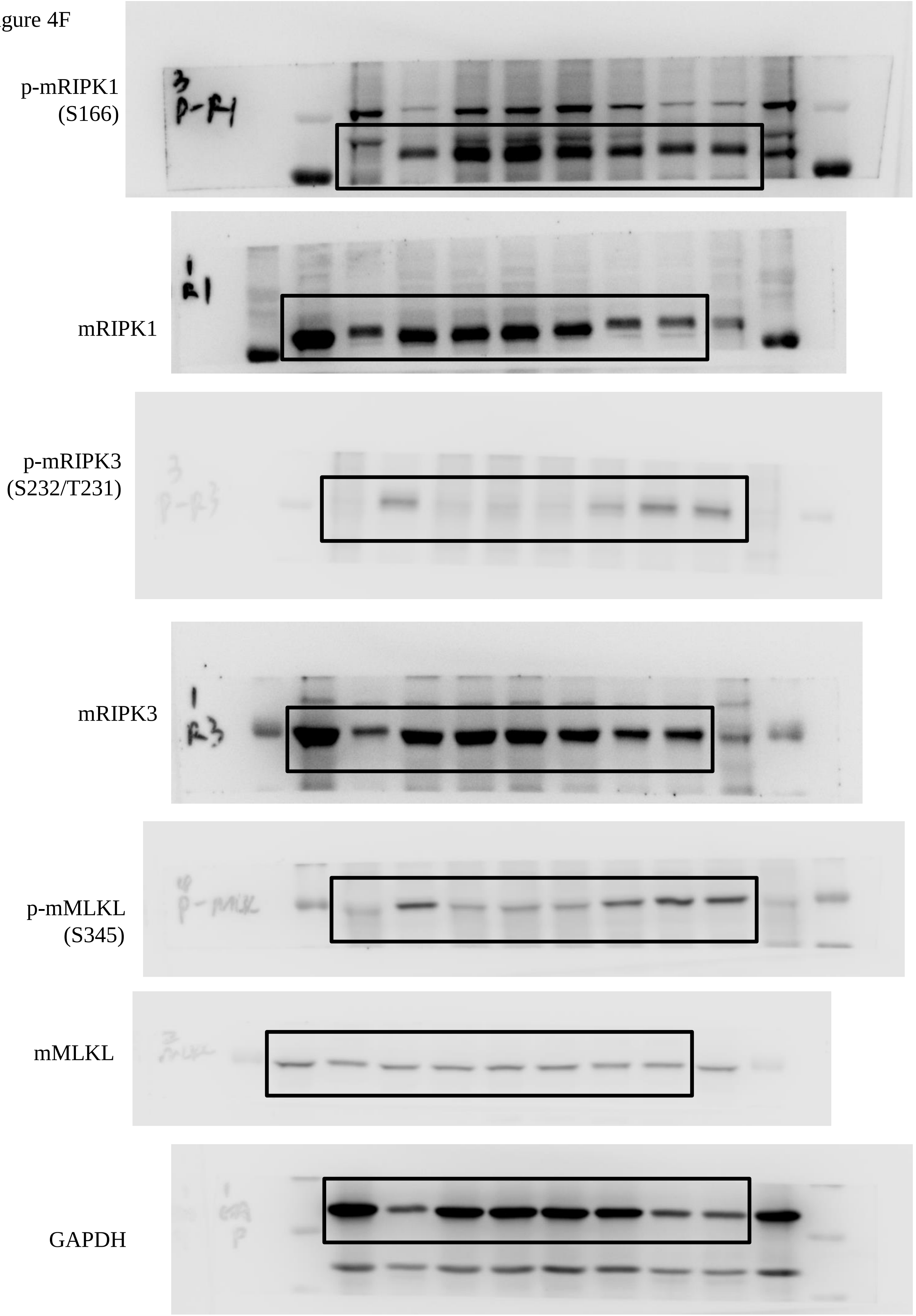
p-mRIPK1
(S166)
mRIPK1
p-mRIPK3
(S232/T231)
mRIPK3
p-mMLKL
(S345)
mMLKL
GAPDH

Figure 5B

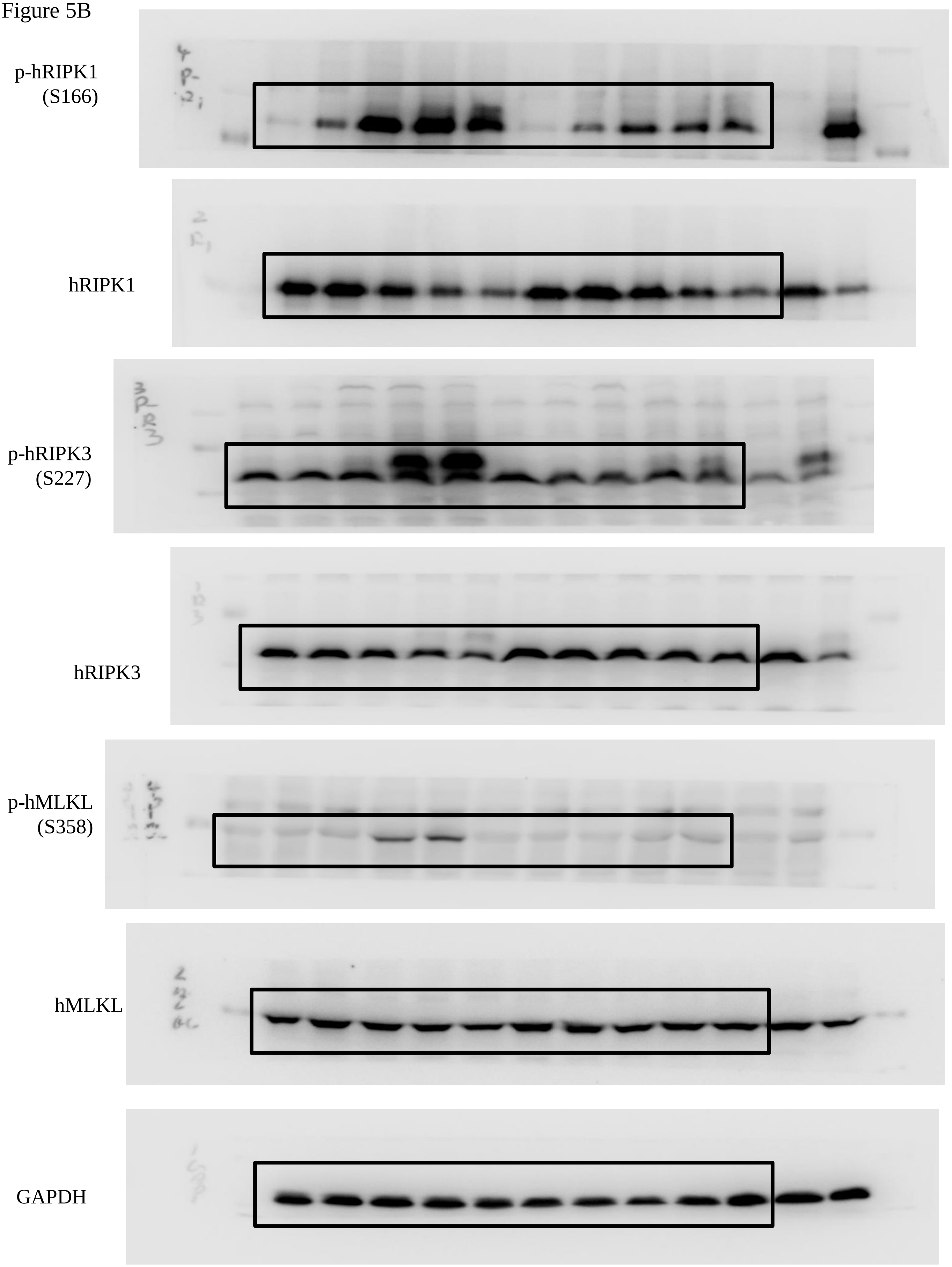

Figure 5C

IP: Flag (mRIPK3)

mRIPK1

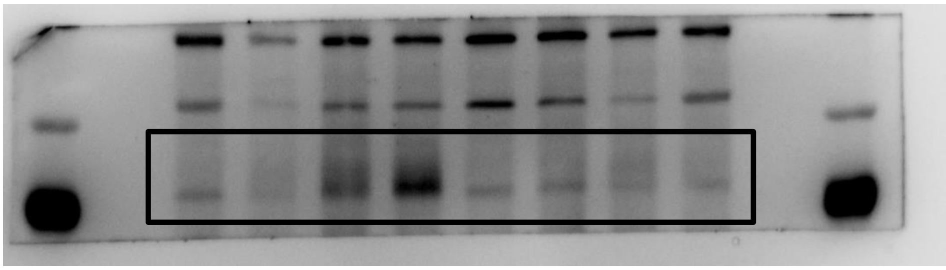

IP: Flag (mRIPK3)

mRIPK3

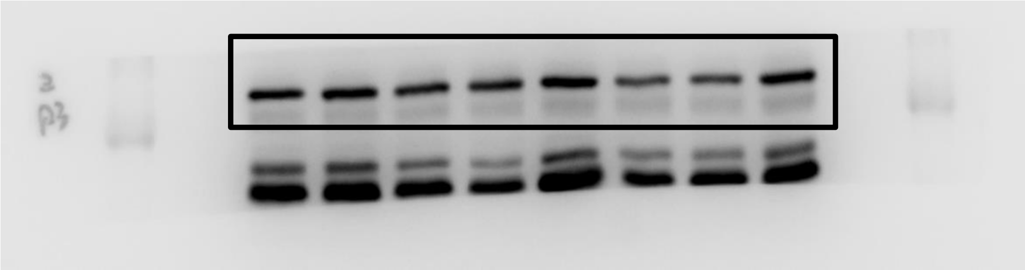

TCL

mRIPK1

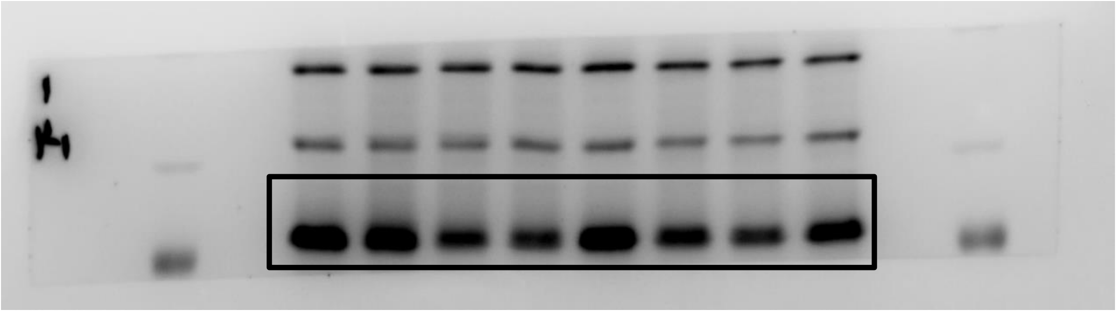

TCL

mRIPK3

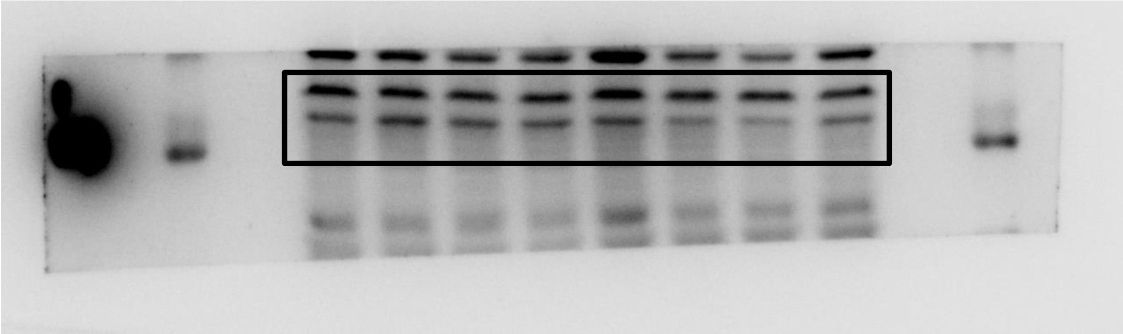

Figure 5D

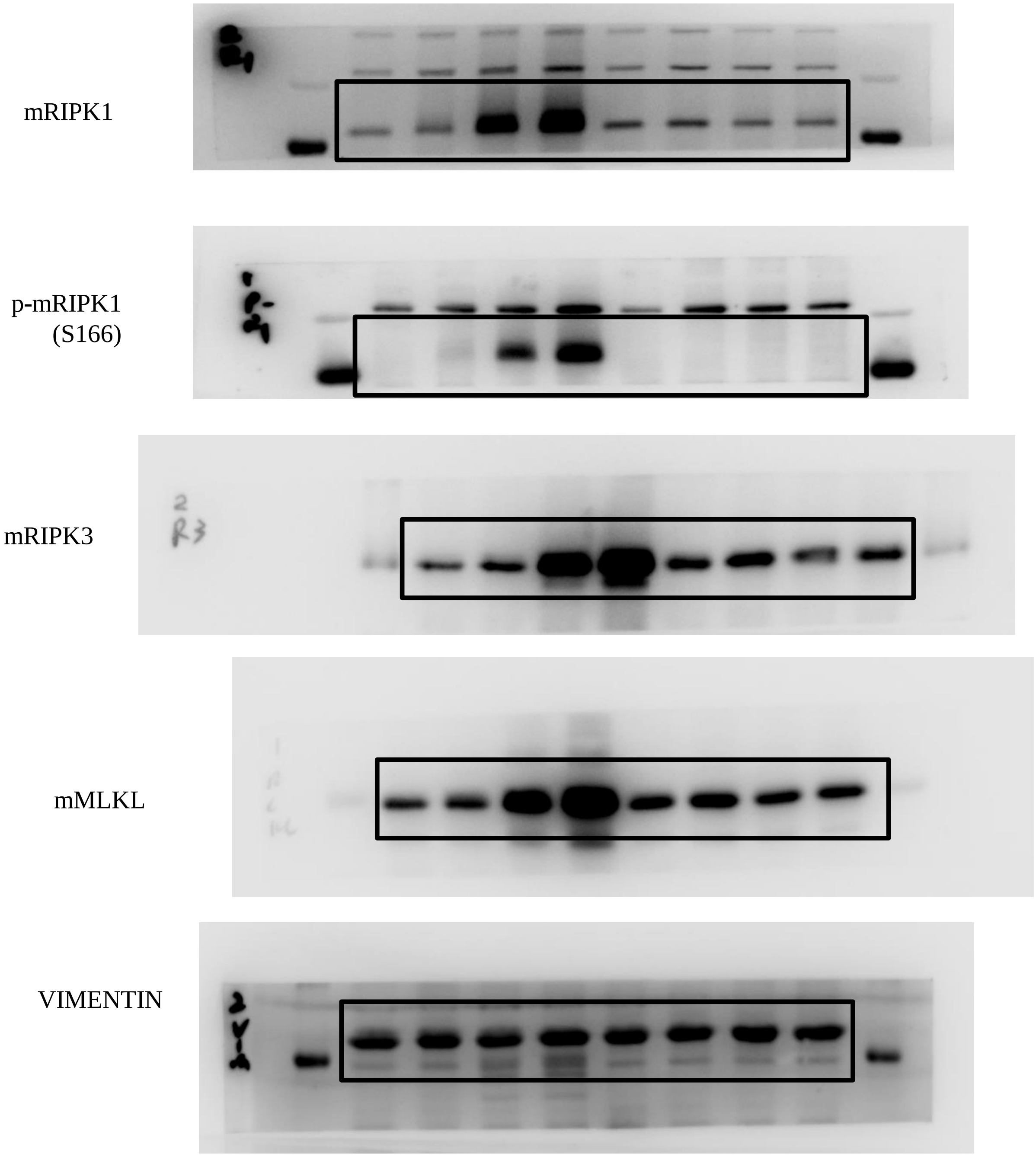

Figure 5F

p-mRIPK1
(S166)

HA

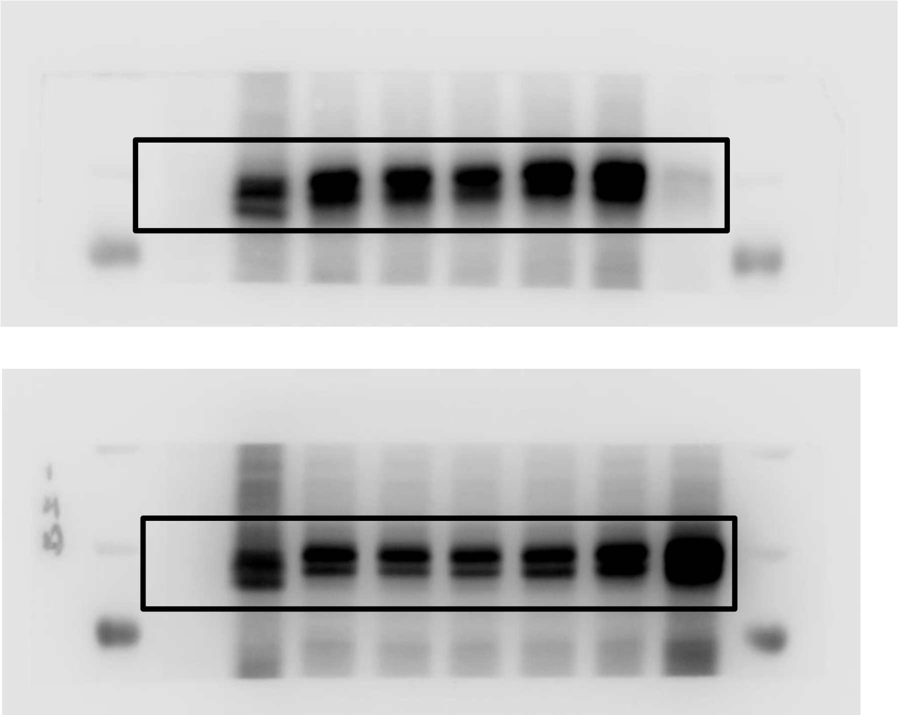

Figure S1A

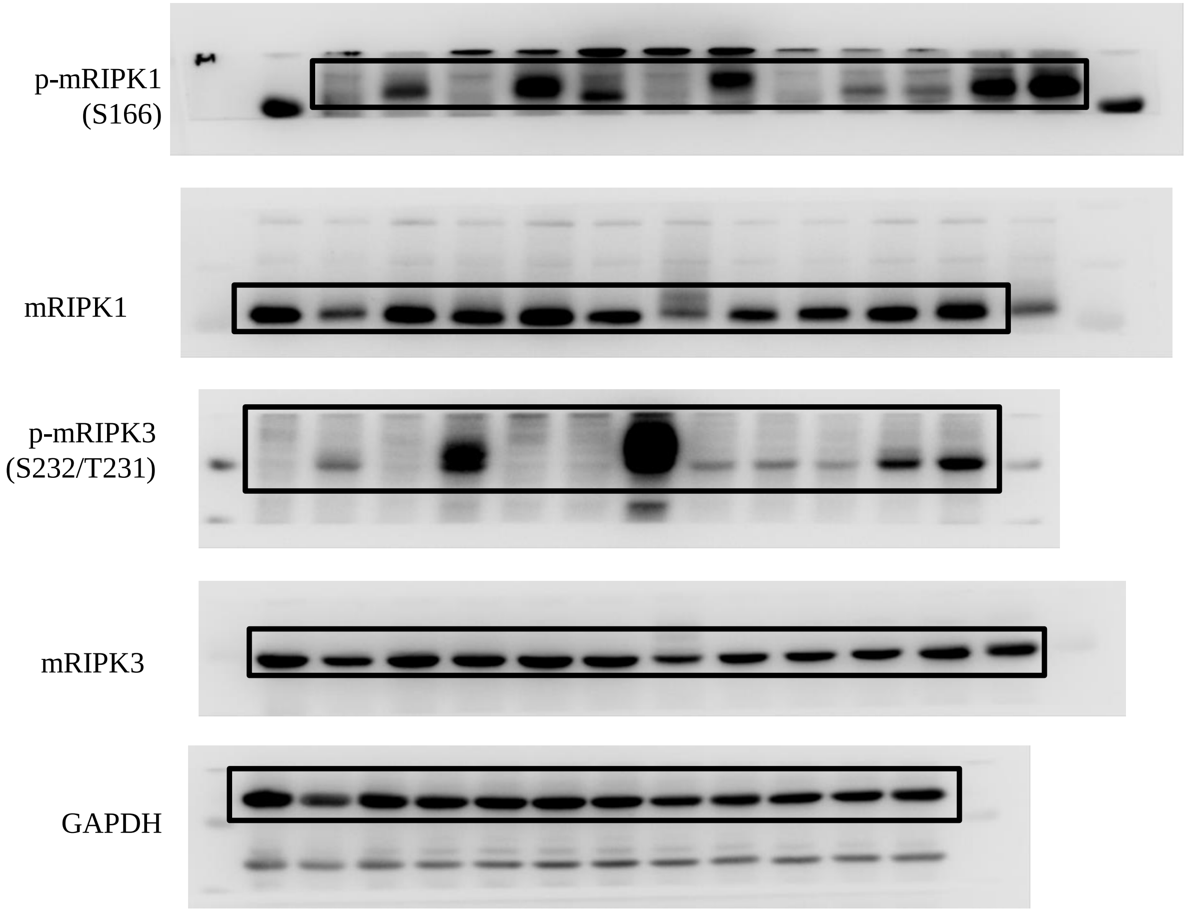

Figure S1B

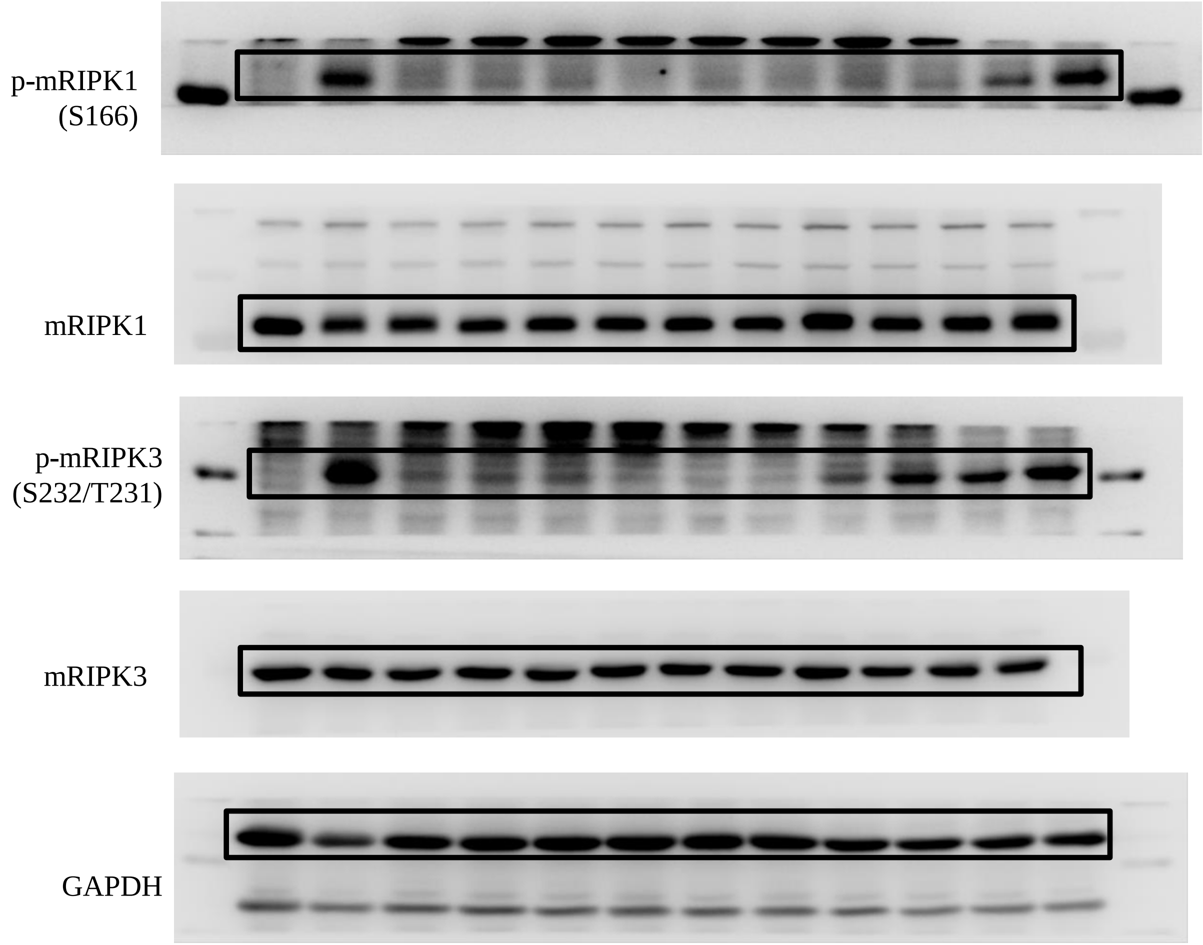

Figure S1C

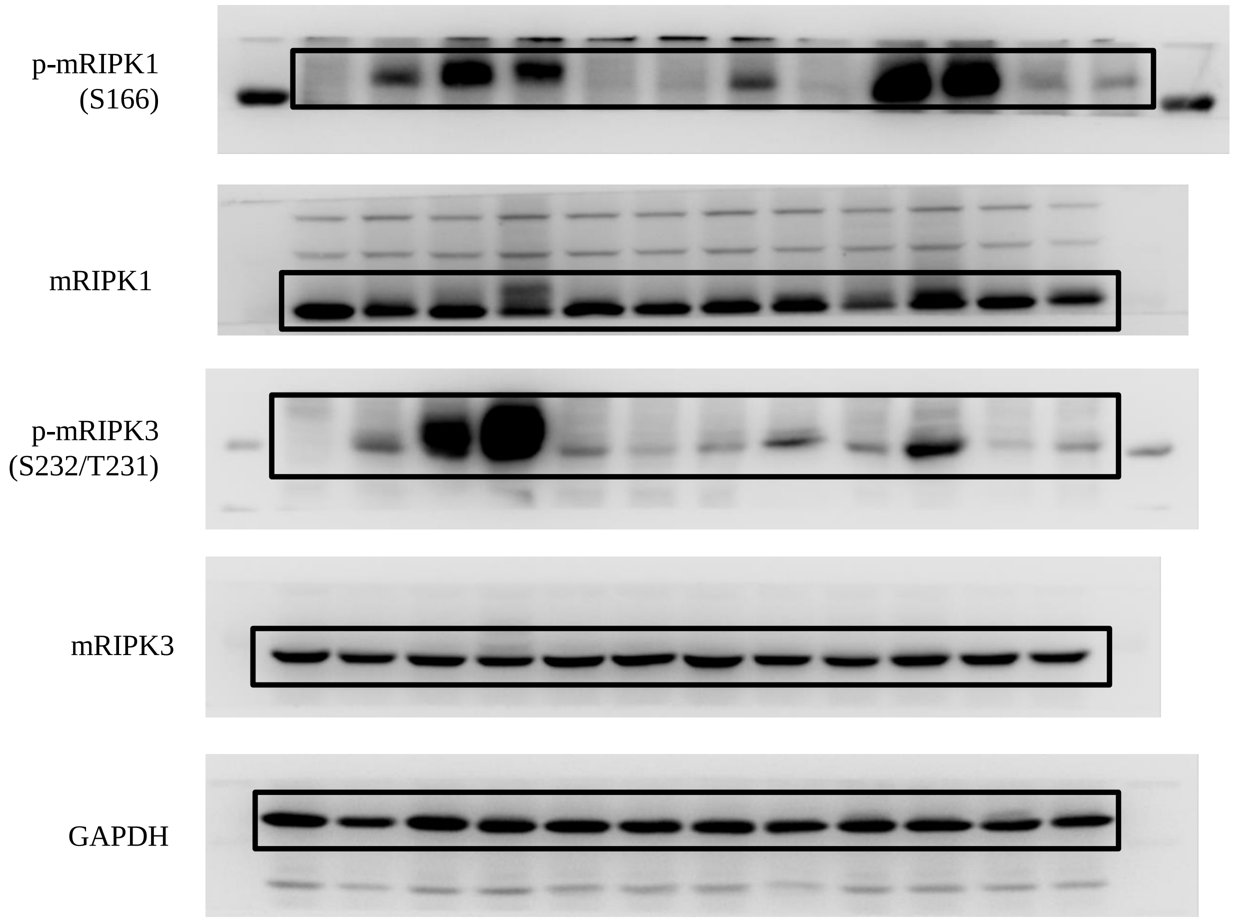

Figure S1D

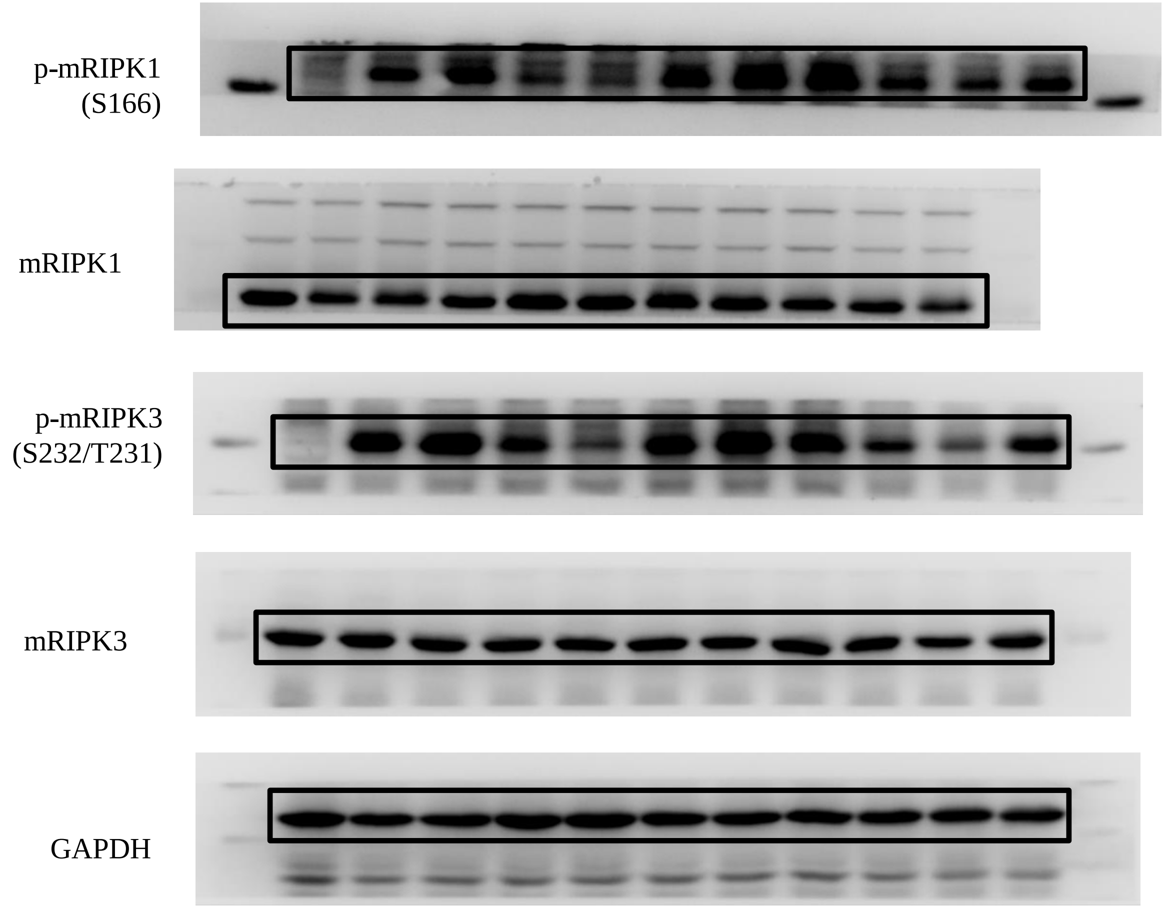

Figure S1E

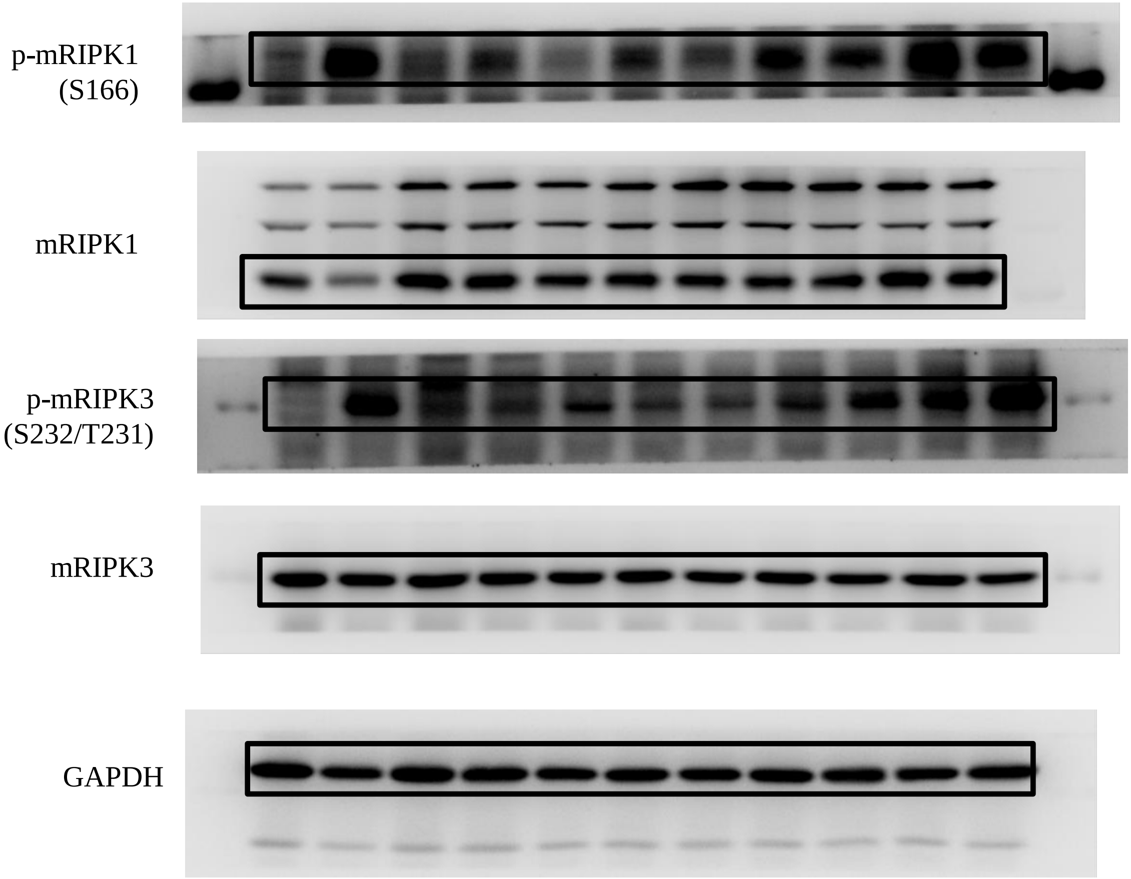

Figure S3

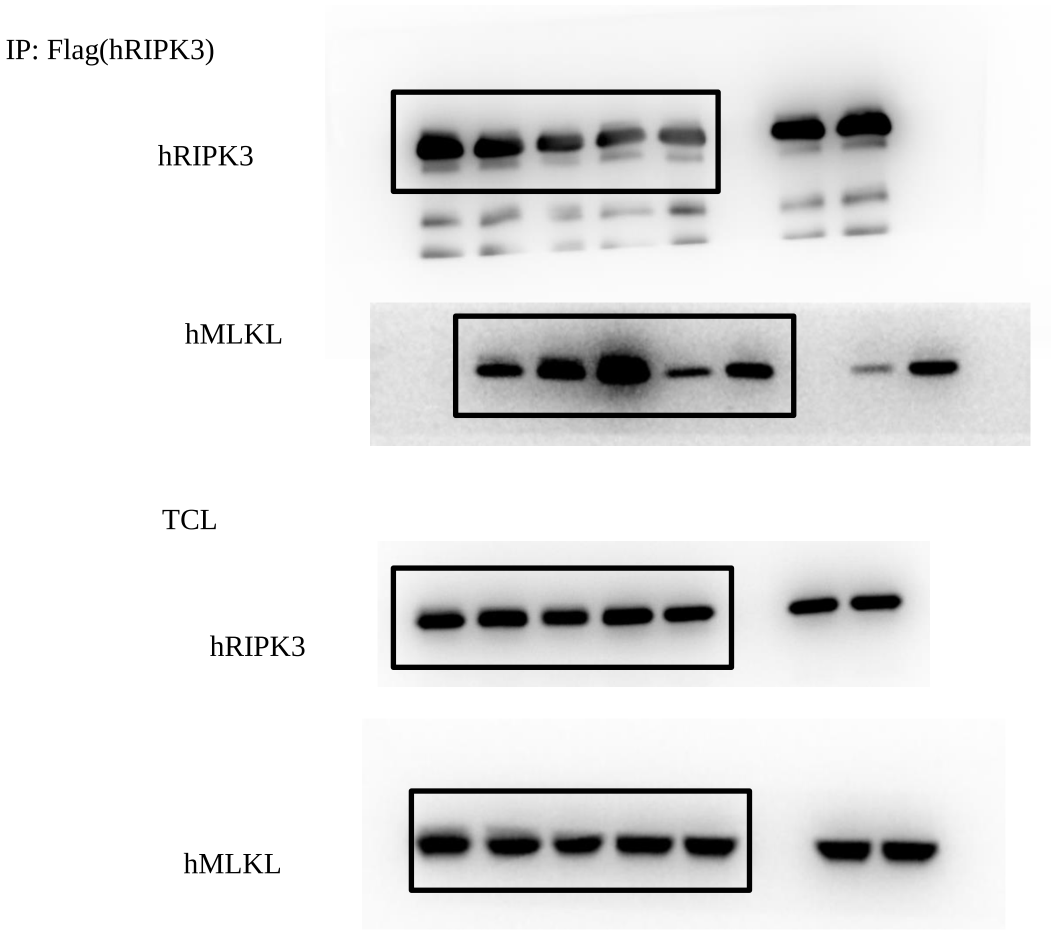

Figure S5

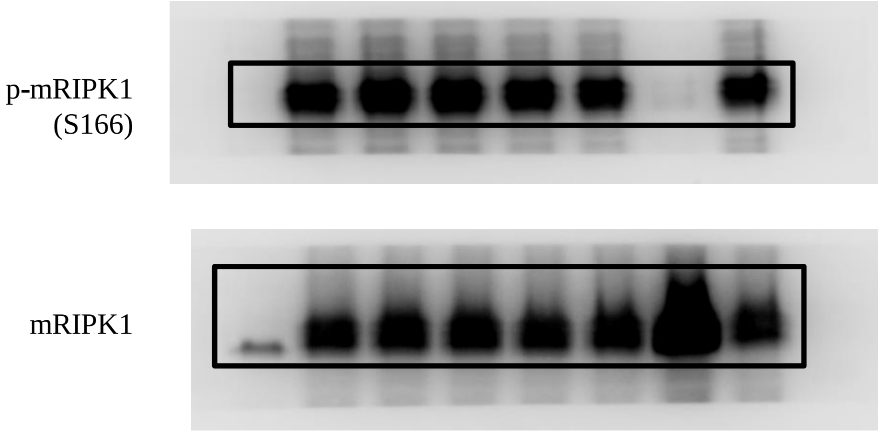

Figure S7

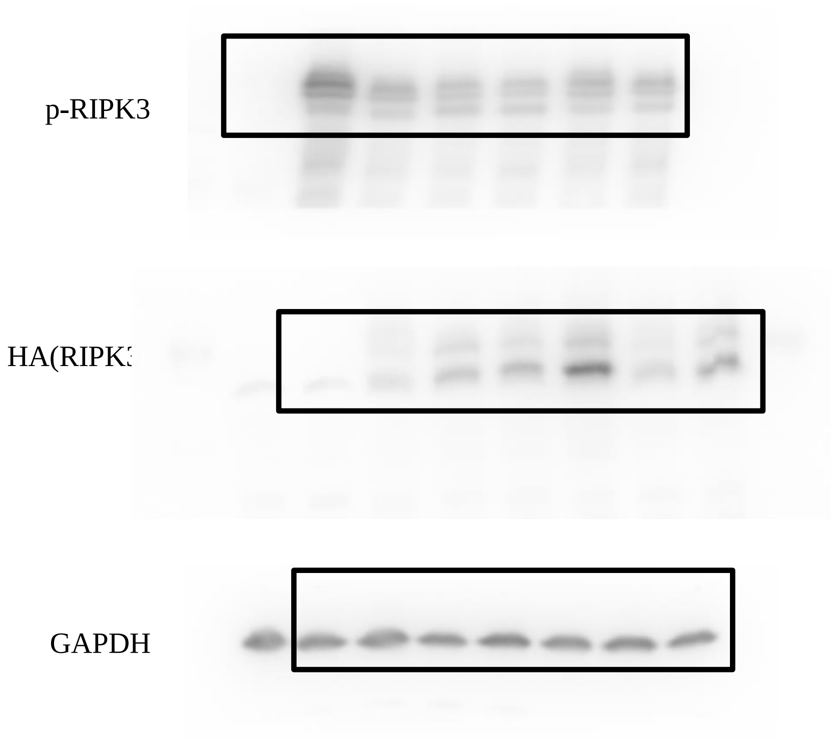

Figure S12B

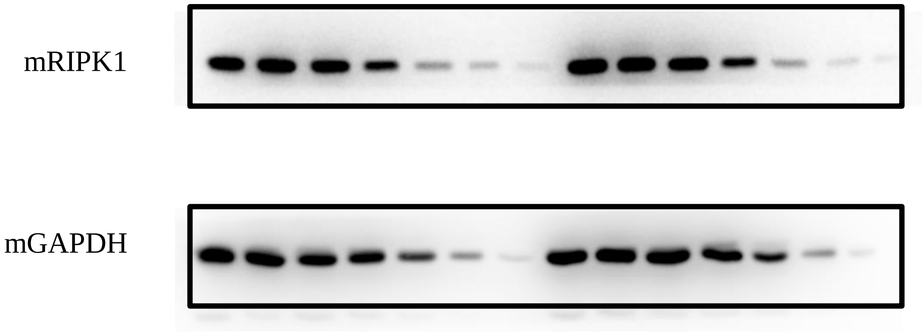

Figure S13A

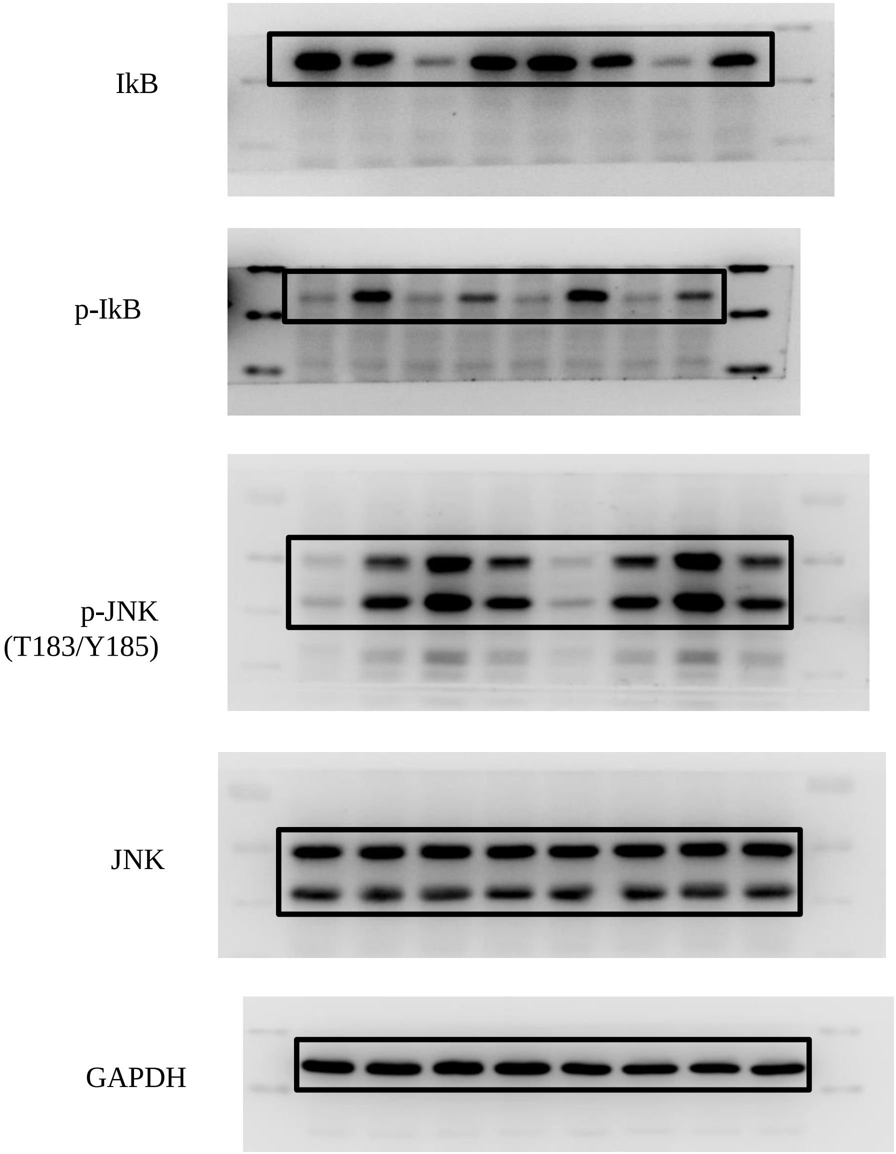

Figure S13B

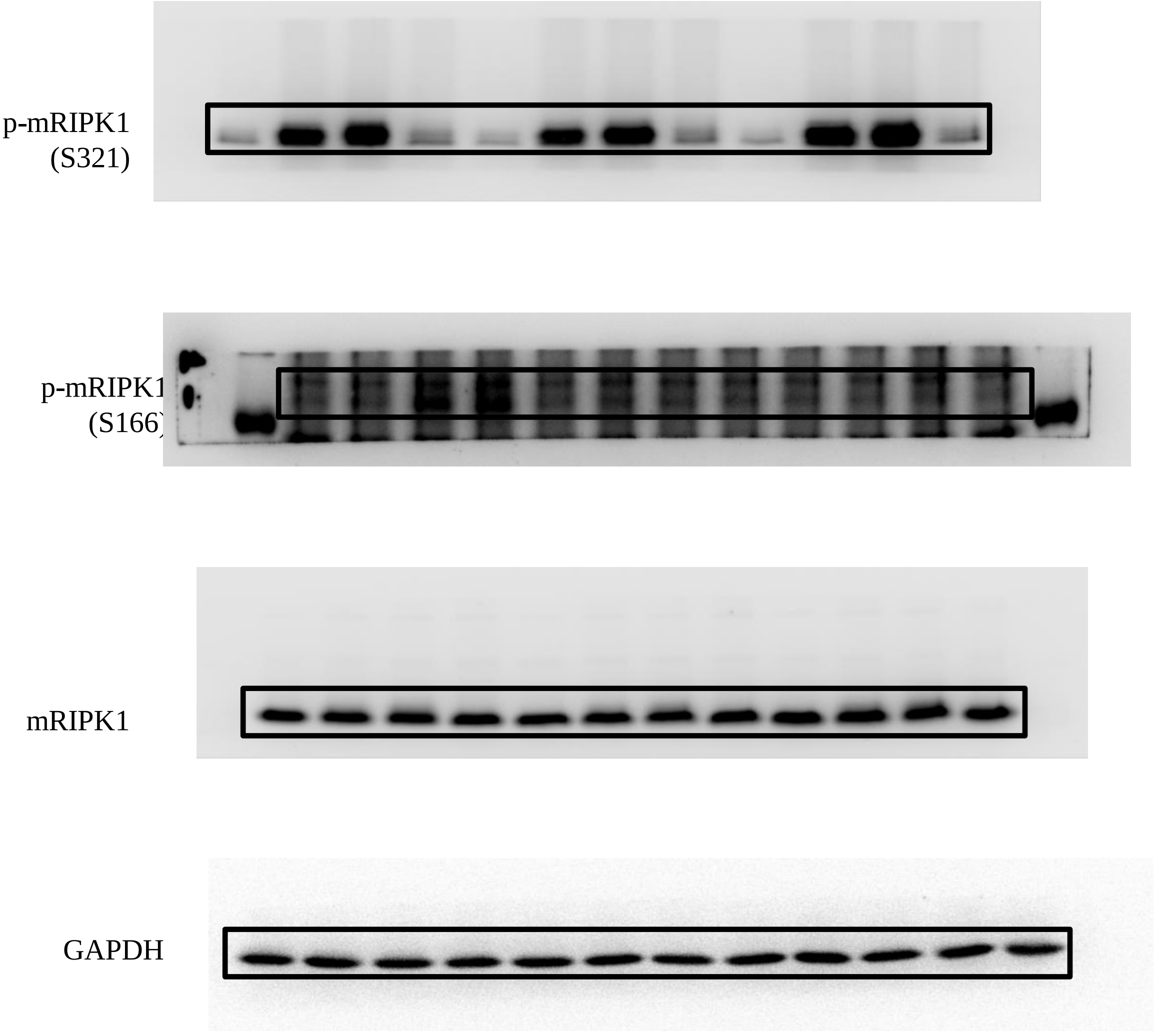

Supplementary Figure 17. Uncropped gel images.
