# Supplementary material for: Repurposing of Ibrutinib and Quizartinib as potent inhibitors of necroptosis
Source: Commun Biol. 2023 Sep 23;6:972. doi: 10.1038/s42003-023-05353-5 (PMC10517925; doi:10.1038/s42003-023-05353-5)
Supplement: Supplementary file 2 — Description of Additional Supplementary Files [file 42003_2023_5353_MOESM2_ESM.pdf]

Description of Additional Supplementary Files

File name: Supplementary Data 1
Description: Compound library

File name: Supplementary Data 2
Description: Necroptosis inhibitors identified in our screening.

File name: Supplementary Data 3
Description: Compounds that reduced necroptosis induced by the dimerization/oligomerization of RIPK1 and RIPK3.

File name: Supplementary Data 4
Description: The source data behind the graphs and charts in the paper.
