# Supplementary material for: Repurposing of Ibrutinib and Quizartinib as potent inhibitors of necroptosis
Source: Commun Biol. 2023 Sep 23;6:972. doi: 10.1038/s42003-023-05353-5 (PMC10517925; doi:10.1038/s42003-023-05353-5)
Supplement: Supplementary file 7 — Reporting Summary [file 42003_2023_5353_MOESM7_ESM.pdf]

nature portfolio

Corresponding author(s): Lanqin Wu, Lisheng Li

Last updated by author(s): Jul 30, 2023

Reporting Summary

Nature Portfolio wishes to improve the reproducibility of the work that we publish. This form provides structure for consistency and transparency in reporting. For further information on Nature Portfolio policies, see our Editorial Policies and the Editorial Policy Checklist.

Statistics

For all statistical analyses, confirm that the following items are present in the figure legend, table legend, main text, or Methods section.

n/a	Confirmed	
☐	☒	The exact sample size (*n*) for each experimental group/condition, given as a discrete number and unit of measurement
☐	☒	A statement on whether measurements were taken from distinct samples or whether the same sample was measured repeatedly
☐	☒	The statistical test(s) used AND whether they are one- or two-sided<br>*Only common tests should be described solely by name; describe more complex techniques in the Methods section.*
☒	☐	A description of all covariates tested
☒	☐	A description of any assumptions or corrections, such as tests of normality and adjustment for multiple comparisons
☐	☒	A full description of the statistical parameters including central tendency (e.g. means) or other basic estimates (e.g. regression coefficient) AND variation (e.g. standard deviation) or associated estimates of uncertainty (e.g. confidence intervals)
☐	☒	For null hypothesis testing, the test statistic (e.g. *F*, *t*, *r*) with confidence intervals, effect sizes, degrees of freedom and *P* value noted<br>*Give P values as exact values whenever suitable.*
☒	☐	For Bayesian analysis, information on the choice of priors and Markov chain Monte Carlo settings
☒	☐	For hierarchical and complex designs, identification of the appropriate level for tests and full reporting of outcomes
☒	☐	Estimates of effect sizes (e.g. Cohen's *d*, Pearson's *r*), indicating how they were calculated

Our web collection on statistics for biologists contains articles on many of the points above.

Software and code

Policy information about availability of computer code

Data collection	Data collection:<br>cell viability/ serum IL-6: Thermo scientific Multiskan Go<br>Kinase activity: TriStar2 S LB 942 Multimode Reader<br>WB: azure biosystems<br>Flow cytometry:FACSCanto II , BD
Data analysis	GraphPad prime 8.0<br>AzureSpot

For manuscripts utilizing custom algorithms or software that are central to the research but not yet described in published literature, software must be made available to editors and reviewers. We strongly encourage code deposition in a community repository (e.g. GitHub). See the Nature Portfolio guidelines for submitting code & software for further information.

Data

Policy information about availability of data

All manuscripts must include a data availability statement. This statement should provide the following information, where applicable:

- Accession codes, unique identifiers, or web links for publicly available datasets
- A description of any restrictions on data availability
- For clinical datasets or third party data, please ensure that the statement adheres to our policy

All datasets generated and analysed during this study are included in this published article and its Supplementary Information files. Additional data are available from the corresponding author on reasonable request.

Human research participants

Policy information about studies involving human research participants and Sex and Gender in Research.

Reporting on sex and gender	NA
Population characteristics	NA
Recruitment	NA
Ethics oversight	NA

Note that full information on the approval of the study protocol must also be provided in the manuscript.

Field-specific reporting

Please select the one below that is the best fit for your research. If you are not sure, read the appropriate sections before making your selection.

☒ Life sciences ☐ Behavioural & social sciences ☐ Ecological, evolutionary & environmental sciences

For a reference copy of the document with all sections, see nature.com/documents/nr-reporting-summary-flat.pdf

Life sciences study design

All studies must disclose on these points even when the disclosure is negative.

Sample size	Sample sizes were determined base on previous studies.
Data exclusions	o data was excluded.
Replication	All experiments were repeated at least twice or three times except the small-compound library screening experiment.
Randomization	Cells or animals were randomly allocated to the experimental groups before treatment.
Blinding	No blinding was performed.

Reporting for specific materials, systems and methods

We require information from authors about some types of materials, experimental systems and methods used in many studies. Here, indicate whether each material, system or method listed is relevant to your study. If you are not sure if a list item applies to your research, read the appropriate section before selecting a response.

Materials & experimental systems

n/a	Involved in the study
☐	☒ Antibodies
☐	☒ Eukaryotic cell lines
☒	☐ Palaeontology and archaeology
☐	☒ Animals and other organisms
☒	☐ Clinical data
☒	☐ Dual use research of concern

Methods

n/a	Involved in the study
☒	☐ ChIP-seq
☐	☒ Flow cytometry
☒	☐ MRI-based neuroimaging

Antibodies

Antibodies used	Rabbit anti-HA (51064-2-AP) and anti-Flag (20543-1-ap), mouse anti-GAPDH (60004-1-Ig), goat anti-mouse (SA00001-1), and goat anti-Rat (SA00001-15) antibodies were purchased from Proteintech company (USA). Goat anti rabbit (A5014) antibodies were purchased from ABclonal company (USA). Rabbit anti JNK (9252T), p-JNK (T183/Y185) (4668T), p-IkB (2859T), p-mRIPK1 (S166) (53286S), p-mRIPK1 (S321) (38662S), hRIPK1 (3493T), hRIPK3 (13526S), hMLKL (14993S), p-hRIPK1 (S166) (65746S), p-hRIPK3 (S227) (93654S), p-hMLKL(S358) (91689S), and mouse anti-IkB (4814T) were purchased from Cell Signaling Technology company (USA). Rabbit anti Vimentin (ab92547), p-mRIPK3 (S232/T231) (ab222320), p-mMLKL (S345) (ab196436), and rat anti-mMLKL (ab243142) were purchased from Abcam company (England). Mouse anti-mRIPK1 (610458) was purchased from BD Biosciences (USA).
Validation	All antibodies were validated by the manufacturers and confirmed by our experiments.

Eukaryotic cell lines

Policy information about cell lines and Sex and Gender in Research

Cell line source(s)	The mouse fibrosarcoma L929 cell line, HEK293T cell line, NIH3T3-RIPK3 cell line and Hela-RIPK3 cell line were kindly provided by prof. Jiahuai Han (Xiamen University, Xiamen).
Authentication	All cell lines used were previously published and well charaterized by many investigators.
Mycoplasma contamination	The cells we used were not contaminate by mycoplasma.
Commonly misidentified lines (See ICLAC register)	Not applicable.

Animals and other research organisms

Policy information about studies involving animals; ARRIVE guidelines recommended for reporting animal research, and Sex and Gender in Research

Laboratory animals	C57BL/6 male mice (8-12 weeks old) were purchased from GemPharmatech Co.,Ltd. (Nanjing, China)
Wild animals	No wild animals were used in this study.
Reporting on sex	Not applicable.
Field-collected samples	Not applicable.
Ethics oversight	All animal experiments were performed in accordance with protocols approved by Institutional Animal Ethical Committee of Fujian Medical University.

Note that full information on the approval of the study protocol must also be provided in the manuscript.

Flow Cytometry

Plots

Confirm that:

- ☐ The axis labels state the marker and fluorochrome used (e.g. CD4-FITC).
- ☐ The axis scales are clearly visible. Include numbers along axes only for bottom left plot of group (a 'group' is an analysis of identical markers).
- ☐ All plots are contour plots with outliers or pseudocolor plots.
- ☒ A numerical value for number of cells or percentage (with statistics) is provided.

Methodology

Sample preparation	L929 cells
Instrument	FACSCanto II, BD
Software	BD FACSDiva Software, Flowjo
Cell population abundance	Not applicable.
Gating strategy	Cell gating: FSC versus SSC<br>Analysis: density plot of SSC versus PI, PI positive area.

☒ Tick this box to confirm that a figure exemplifying the gating strategy is provided in the Supplementary Information.
